# Hormonal status and age differentially affect tolerance to the disruptive effects of delta-9-tetrahydrocannabinol (Δ^9^-THC) on learning in female rats

**DOI:** 10.3389/fphar.2015.00133

**Published:** 2015-07-02

**Authors:** Peter J. Winsauer, Catalin M. Filipeanu, Peter F. Weed, Jessie L. Sutton

**Affiliations:** ^1^Department of Pharmacology and Experimental Therapeutics, Louisiana State University Health Sciences Center New OrleansNew Orleans, LA, USA; ^2^Alcohol and Drug Abuse Center of Excellence, Louisiana State University Health Sciences Center New OrleansNew Orleans, LA, USA; ^3^Department of Pharmacology, Howard University College of MedicineWashington, DC, USA

**Keywords:** chronic Δ^9^-tetrahydrocannabinol, age of initiation, ovariectomy, learning, cannabinoid receptors, rat

## Abstract

The effects of hormone status and age on the development of tolerance to Δ^9^-THC were assessed in sham-operated (intact) or ovariectomized (OVX) female rats that received either intraperitoneal saline or 5.6 mg/kg of Δ^9^-THC daily from postnatal day (PD) 75–180 (early adulthood onward) or PD 35–140 (adolescence onward). During this time, the four groups for each age (i.e., intact/saline, intact/THC, OVX/saline, and OVX/THC) were trained in a learning and performance procedure and dose-effect curves were established for Δ^9^-THC (0.56–56 mg/kg) and the cannabinoid type-1 receptor (CB1R) antagonist rimonabant (0.32–10 mg/kg). Despite the persistence of small rate-decreasing and error-increasing effects in intact and OVX females from both ages during chronic Δ^9^-THC, all of the Δ^9^-THC groups developed tolerance. However, the magnitude of tolerance, as well as the effect of hormone status, varied with the age at which chronic Δ^9^-THC was initiated. There was no evidence of dependence in any of the groups. Hippocampal protein expression of CB1R, AHA1 (a co-chaperone of CB1R) and HSP90β (a molecular chaperone modulated by AHA-1) was affected more by OVX than chronic Δ^9^-THC; striatal protein expression was not consistently affected by either manipulation. Hippocampal brain-derived neurotrophic factor expression varied with age, hormone status, and chronic treatment. Thus, hormonal status differentially affects the development of tolerance to the disruptive effects of delta-9-tetrahydrocannabinol (Δ^9^-THC) on learning and performance behavior in adolescent, but not adult, female rats. These factors and their interactions also differentially affect cannabinoid signaling proteins in the hippocampus and striatum, and ultimately, neural plasticity.

## Introduction

Research over the past decade or so has highlighted the fact that the cannabinoids can produce sexually dimorphic effects on learning and memory, as well as on a variety of behavioral processes and physiological systems. These sexually dimorphic effects most likely have their origin during adolescence when the sex hormones begin to exert their organizational and activational effects (Arnold and Breedlove, 1985; Sisk and Zehr, 2005). Thus, any disruption of these organizational and activational effects, such as a reduction of the sex hormones or excessive exogenous activation of specific neurotransmitter systems, could produce long-term disruptions of the systems or behaviors mediated by those hormones. This may be especially true for the cannabinoid system, as there is a growing literature on this system’s age-dependent, bidirectional interaction with the sex hormones, particularly in females (Gorzalka and Dang, 2012). For example, we have found that estradiol capsules implanted in ovariectomized (OVX) females significantly attenuated both the rate-decreasing and error-increasing effects of Δ^9^-THC in mature females responding in an operant task where subjects learned (acquired) a different sequence of responses each session (Daniel et al., 2002). However, this effect of estradiol was later shown to be age-dependent, as intact females were found to be more sensitive than OVX females to the disruptions of rate and accuracy produced by 40 days of chronic Δ^9^-THC administration during adolescence (Winsauer et al., 2011a). The increased sensitivity of intact females compared to OVX females was also evident when 40 days of chronic Δ^9^-THC administration was initiated during early adulthood (Winsauer and Sutton, 2014) – suggesting that estradiol may not be ‘protective’ until long after its organizational and activational effects have been established.

The purpose of the present study was to determine whether the initiation of chronic Δ^9^-THC administration during either adolescence or early adulthood would affect the development of tolerance to its disruptive effects on the operant procedure of learning (acquisition) and performance behavior that was used in the aforementioned studies. In this procedure, subjects both acquire a new sequence of responses each session (i.e., repeated acquisition) and emit a well-rehearsed sequence of responses (performance). Such baselines of operant behavior have several advantages. One advantage is that responding in the performance component can serve as a behavioral control for the non-specific effects of either drug or hormone status. Another advantage is that having a stable baseline of repeated acquisition behavior avoids the confound associated with “learning to learn” (Harlow, 1949), and allows for the assessment of learning over a long period of time. The major difference, however, between the present study and previous studies was that there was no drug-free period after chronic Δ^9^-THC was initiated, which for the adolescents resulted in almost life-long Δ^9^-THC administration.

Few human or animal studies have taken this type of longitudinal approach, or administered chronic Δ^9^-THC for this duration (e.g., Fehr et al., 1976; Ali et al., 1989; Winsauer et al., 2011b). Most of the prospective human studies are those that have followed self-reported users over long periods of time and then tested them periodically (Brook et al., 2008; Meier et al., 2012) or examined the acute effects of Δ^9^-THC in frequent or long-time users as compared to non-users (Solowij et al., 1991; Kirk and de, 1999; Pope et al., 2003; Jacobsen et al., 2004; Kanayama et al., 2004; Lane et al., 2007). Brook et al. (2008), for example, followed 749 participants over a period of 13 years and chronicled any emergent health problems by interviewing them at mean ages of 14, 16, 22, and 27 years. Of note, marijuana use was significantly associated with respiratory problems, general malaise, neurocognitive problems, and lower academic achievement and functioning. Similar findings were also obtained from the Dunedin study, a prospective study of 1,037 individuals followed from birth to age 38. Interviews for this study occurred at 18, 21, 26, 32, and 38 years of age, and neuropsychological testing was conducted at age 13 (Meier et al., 2012). Although there were concerns that not enough weight was given to the importance of socioeconomic status in this study (cf. Rogeberg, 2013), the results indicated that there may be residual effects as ‘cessation of cannabis use did not fully restore neuropsychological functioning among those that started during adolescence.’

Foremost among the findings from longitudinal studies such as the Dunedin study are that executive functioning (Meier et al., 2012) and processing speed or reaction time (Mendhiratta et al., 1988; Wilson et al., 1994; Hunault et al., 2009) tend to be the most susceptible to the long-term marijuana use and that disruptions of these neurocognitive processes can eventually lead to motivational and academic difficulties (for reviews, see Lynskey and Hall, 2000; Gruber and Pope, 2002; Volkow et al., 2014). These findings have also been bolstered by the human studies examining the acute and chronic effects of Δ^9^-THC, which have documented Δ^9^-THC-induced disruptions in a number of domains of neuropsychological functioning. In an often cited paper, Ehrenreich et al. (1999) found impaired reaction time for a visual scanning task, but no effect on several other attentional functions. Pope et al. (2003) found that there was a marked difference in verbal intelligence quotient between early onset users and late-onset users. Deficits in working memory have also featured prominently in the effects reported by a number of investigators (Wilson et al., 1994; Jacobsen et al., 2004; Kanayama et al., 2004; Hunault et al., 2009).

Another important aspect of the present study was the assessment of the long-term effects of Δ^9^-THC administration on cannabinoid signaling in the hippocampus and striatum. These two brain regions were found by us (Winsauer et al., 2011a) and others (Breivogel et al., 1999; Rubino et al., 2000a; McKinney et al., 2008; Burston et al., 2010; Hirvonen et al., 2012) to be directly affected by long-term chronic Δ^9^-THC administration during adolescence and to be regions where there was clear evidence of an interaction between cannabinoid administration and ovarian hormones. In our study, a period of chronic Δ^9^-THC during adolescence increased cannabinoid type-1 receptors (CB1Rs) in the hippocampus of intact female rats, and had no effect on CB1R in striatum, although OVX reduced CB1R irrespective of chronic Δ^9^-THC administration in this region. Due to effects such as these, the present study examined the expression of CB1R and the expression of AHA1 and HSP90β in both hippocampus and striatum following chronic Δ^9^-THC. The role of AHA1 in cannabinoid signaling was first pointed out by Filipeanu et al. (2011), and they showed that its expression *in vivo* was increased by both chronic Δ^9^-THC and OVX. Further, altering AHA1 expression *in vitro* demonstrated that this protein was a chaperone of CB1R (but not of CB2R), as overexpression of AHA1 was found to enhance plasma membrane levels of CB1R, enhance CB1R-mediated reductions in cyclic adenosine monophosphate (cAMP), and increase Δ^9^-THC-mediated activation of MAPK through CB1R. Before this study, the only known role of AHA1 was as a co-activator of HSP90, a heat-shock protein involved in protein folding, traffic, and degradation (Wandinger et al., 2008). However, HSP90 levels in the cerebellum were not changed by chronic Δ^9^-THC administration during adolescence. The research plan for the present study was to extend those observations to the hippocampus and striatum, areas that are also important contributors to learning and performance behavior. Further, no studies to our knowledge have investigated the effects of chronic Δ^9^-THC and hormone status on these proteins in females for the duration used in this study. Brain-derived neurotrophic factor (BDNF) expression was of interest because of all of the data indicating that there is impaired neuronal plasticity in the hippocampus when chronic Δ^9^-THC is initiated during adolescence (Volkow et al., 2014), and both chronic Δ^9^-THC and estrogen interact with this trophic factor (Butovsky et al., 2005; Scharfman and Maclusky, 2005; Maj et al., 2007; De Chiara et al., 2010).

## Materials and Methods

### Subjects

A total of 71 female Long-Evans rats served as subjects. With respect to hormonal status, 35 were OVX, whereas the other 36 underwent sham surgery (intact). These two hormone groups were then further subdivided into groups that received either chronic saline or Δ^9^-THC, yielding four treatment groups with respect to hormonal status and chronic administration (i.e., intact/saline, intact/THC, OVX/saline, and OVX/THC). An additional variable was whether chronic administration of saline or Δ^9^-THC began during adolescence or early adulthood. For the behavioral experiments, 24 females received saline or Δ^9^-THC from adolescence [postnatal day (PD) 35] onward, whereas another 24 received saline or Δ^9^-THC from early adulthood (PD 75) onward. The remaining 23 females received all of the experimental manipulations, but were used solely for analyses of pharmacodynamics variables (e.g., receptor levels).

The female rats were purchased from a commercial vendor (Harlan Sprague Dawley, Indianapolis, IN, USA) in two cohorts as pups and arrived at the Animal Care facility on PD 21. Pups were group housed until PD 30 and provided a standard diet of rodent chow (Rodent Diet 5001, PMI Inc., St. Louis, MO, USA) and water *ad libitum*. On PD 30, all the subjects underwent either OVX or a sham surgery. Following these procedures, the subjects were individually housed in polypropylene plastic cages with hardwood chip bedding. Food restriction was also instituted at this time to maintain the compatibility of the treated groups, as OVX females have been shown to gain weight at a faster rate than intact females (e.g., Winsauer et al., 2011a); in this case, subjects were maintained at approximately 90% of their free-feeding weights while allowing for a minimum gain of 5–10 grams per week to control for normal growth.

The colony room was maintained at 21 ± 2°C with 50 ± 10% relative humidity on a 14L:10D light/dark cycle (lights on 06:00 h, lights off 20:00 h) throughout testing. All subjects were also maintained in accordance with the Institutional Animal Care and Use Committee, Louisiana State University Health Sciences Center, and in compliance with the recommendations of the National Research Council in the Guide for the Care and Use of Laboratory Animals (National Research Council, 1996).

### Adolescent Ovariectomies

As indicated above, 35 subjects were OVX while under general anesthesia induced by intraperitoneal (i.p.) injection of ketamine (50 mg/kg) and xylazine (10 mg/kg), as detailed in Winsauer et al. (2011a). The remaining females underwent sham surgeries as a control for the ovariectomy. During sham surgeries, the subjects were anesthetized with ketamine/xylazine, shaved, and bilateral flank incisions were made, but the ovaries were not isolated or removed. Female rats generally recover from both procedures fully within 2 days after surgery, and this was evident from their activity levels, food and water intake, and urination and defecation.

### Chronic Administration of Saline or Δ^9^-THC

Whether chronic administration began on PD 35 or PD 75, the chronic regimen consisted of either a single, daily injection of saline or 0.56 mg/kg of Δ^9^-THC. This injection was administered at the beginning of each day, 7 days per week, with the exception of a brief period (∼21 days) for a large number of adolescents and a small number of adults to facilitate training the behavioral groups. During this brief period, injections were administered in the early afternoon after their training sessions. Throughout the rest of the study (164 days for the adolescents and 124 days for the adults), injections were administered in the morning prior to testing the two groups of 12 animals, which had subjects from each age and treatment group to control for the time from the chronic injection.

The Δ^9^-THC was obtained from the National Institute on Drug Abuse (Research Technical Branch, Rockville, MD, USA), and arrived in a 100% ethanol solution at a concentration of either 100 or 200 mg/ml. These concentrations were then partitioned into smaller aliquots (e.g., 50 mg), lyophilized by high-speed vacuum, and then stored at -20°C. When needed, the aliquots of Δ^9^-THC were reconstituted for injection as an emulsion using ethanol, emulphor (Alkamuls EL-620, Rhodia, Inc., Cranbury, NJ, USA), and saline in a proportion of 1:1:18, respectively. The volume for both saline and Δ^9^-THC injections was 0.1 ml/100 g body weight. Given this injection volume, the percentage of ethanol (5%) in the vehicle had no behavioral consequences. For example, the injection volume for a 260 g rat was 0.26 ml, and 5% of 0.26 ml is 0.013 ml, which equals 0.01 g of ethanol when 0.013 ml is multiplied by the density of ethanol at standard temperature and pressure (0.789 g/ml). This 0.01 g divided by the weight of the rat (0.26 kg) yields a 0.04-g/kg dose of ethanol, which is substantially below an effective dose for ethanol.

### Apparatus for Behavioral Testing

Twelve identical modular test chambers (Coulbourn Instruments, Allentown, PA, USA, Model E10-10TC) configured specifically for rodents were used, and have been described in detail previously (Winsauer et al., 2011a). Briefly, located on the front wall of each chamber were a houselight, speaker, auditory feedback relay, pellet trough (5.5 cm above the floor and centered), and three response keys aligned horizontally (8 cm apart, center to center, and 14.5 cm above the floor). Each response key could be transilluminated by three Sylvania 28PSB indicator lamps, one with a red plastic cap, one with a yellow cap, and one with a green cap. Correct responses produced an audible click of the feedback relay. Each chamber was enclosed within a sound-attenuating cubicle equipped with a fan for ventilation and white noise to mask extraneous sounds. All test chambers were connected to a computer programmed in MED-PC for Windows, Version IV (MED Associates, Inc., St. Albans, VT, USA), and to cumulative recorders (Gerbrands, Arlington, MA, USA) located within the same room.

### Behavioral Procedure

**FIGURE 1:**
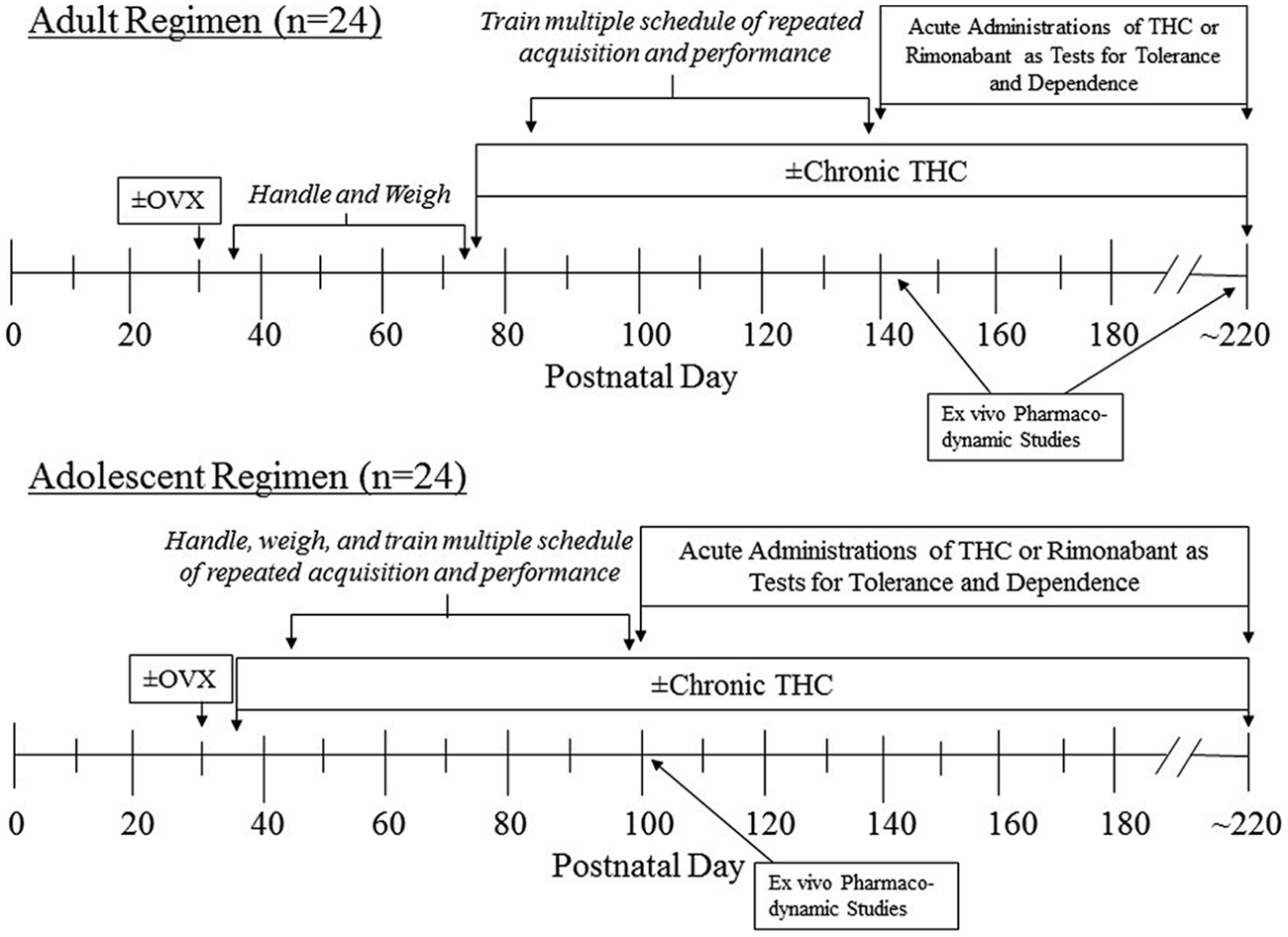
**Timeline of manipulations for the two age groups of female subjects that were administered chronic Δ^9^-THC after either ovariectomy (OVX) or a sham operation**.

As diagramed in **Figure 1**, training both the adolescent and adult groups to respond under the behavioral procedure began 10 days after the onset of chronic saline or Δ^9^-THC administration, which was PD 45 for the adolescents and PD 85 for the adults. Because training was initiated later for the adults, the adult groups were handled and weighed each day to maintain some compatibility between the adolescents and the adults in terms of their interaction with the experimenter. This amounted to acclimating them to being held by the experimenter for increasing durations, and placing them in an empty cage located on an electronic balance to obtain their daily weight.

Preliminary training of the subjects to respond (i.e., nose poke) on a key in the apparatus, and to acquire a three-response sequence on a daily basis (i.e., repeated acquisition), has been described previously (Gerak et al., 2004). Ultimately, the goal of this multiple-schedule procedure was to have the subjects acquire a different three-response sequence each session in the acquisition components and respond on an invariant or well-rehearsed sequence in the performance components. After this preliminary training was completed, a multiple schedule with repeated acquisition and performance components was instituted. Whether in the acquisition or performance component, each subject’s task was to respond on the correct key in the presence of one of three stimulus colors (e.g., keys green, center correct; keys red, left correct; keys yellow, right correct). When the response sequence was completed by emitting three correct responses (i.e., one correct response in the presence of each color), the key lights were extinguished and the stimulus light in the pellet trough was illuminated for 0.4 s. Subsequently, the response keys were illuminated with the first stimulus (i.e., green) and the sequence was reset. Sequence completions were maintained by food presentation under a second-order fixed-ratio (FR) three schedule, as every three completions of the sequence resulted in the presentation of a 45-mg food pellet (Purina Mills TestDiet, Richmond, VA, USA). When rats responded on an incorrect key (in the example above, the left or right key when the green lights were illuminated), the error was followed by a 5-s period in which the keylights were extinguished and responses had no programmed consequence (i.e., a timeout).

To establish a steady state of repeated acquisition, the sequence in the acquisition components was changed from session to session (i.e., daily). An example of sequences for five consecutive sessions was: C–L–R, L–R–C, C–R–L, R–L–C, and L–C–R. The sequences were carefully selected to be equivalent in several ways, and there were restrictions on their ordering across sessions. Briefly, each sequence was scheduled with equal frequency and consecutive correct responses within a sequence were scheduled on different keys. Occasionally, a correct sequence position for a given color was the same for two consecutive sessions (as in the list of sequences above, L–R–C and C–R–L).

During performance components, the houselight and the response keys were illuminated. The houselight served as a discriminative stimulus for responding during this component, and unlike the acquisition component, the sequence in this component remained the same from session to session (i.e., R-C-L). Experimental sessions always began with an acquisition component, which then alternated with a performance component after 40 reinforcers or 20 min, whichever occurred first. Each session terminated after 150 reinforcers or 80 min, whichever occurred first. Throughout testing, sessions were generally conducted 5 days per week, Monday through Friday.

### Dose-Effect Determinations of Δ^9^-THC or Rimonabant during Chronic Δ^9^-THC or Saline Administration

When the response rate and the percentage of errors varied by less than 20% of the mean for 10 consecutive days, dose-effect curves for Δ^9^-THC and rimonanbant were determined by replacing the chronic saline or Δ^9^-THC injection with a different dose every 3 or 4 days. The presession administration time for both drugs was always 30 min, and the doses tested ranged from ineffective to one that substantially decreased overall response rate or eliminated responding entirely. As a control for these acute Δ^9^-THC or rimonabant injections, saline or vehicle injections were also administered occasionally, 30 min prior to the start of the session. The vehicle for rimonabant was comprised of ethanol (33.33%), emulphor (33.33%), and saline (33.33%).

### Western Blotting

Subjects from the groups for the pharmacodynamics studies were sacrificed prior to the determination of the Δ^9^-THC and rimonabant dose-effect curves in the behavioral groups to yield an understanding of the neuronal protein levels that resulted from the chronic dose alone. The behavioral groups were sacrificed after the determination of dose-effect curves for both Δ^9^-THC and rimonabant in each treatment group. At the respective time points, subjects from each treatment group were sacrificed in pairs for analysis of hippocampal protein expression. To control for the effects of circulating levels of endogenous hormones across the estrous cycle, each gonadally intact female was sacrificed with one OVX female from the respective chronic groups (saline or Δ^9^-THC) when the gonadally intact member of the pair was in proestrus (i.e., every intact/THC was paired with a OVX/THC, and every intact/saline was paired with a OVX/saline). The hippocampus and striatum were separated from the rest of the brain using the procedure described by Glowinski and Iversen (1966), and levels of CB-1, Heat-shock protein 90 beta (HSP90β), heat-shock 90 kDA protein ATPase homolog 1 (AHA-1), and BDNF were determined by western blot analyses of homogenate. Western-blot analysis of protein expression was carried out as previously described (Winsauer et al., 2011a, 2012). Briefly, the samples were separated by 10% sodium dodecyl sulfate-polyacrylamide gel electrophoresis (SDS-PAGE), followed by transfer onto polyvinylidene fluoride (PDVF) membranes. The primary antibodies used were anti-CB1 receptor (polyclonal antibody, item #101500, Cayman Chemical, Ann Arbor, MI, USA), anti-AHA1 (monoclonal antibody, catalog number H00010598-M01, AbNova, Taipei City, Taiwan), anti-HSP90β (monoclonal antibody, item K3705, Enzo Life Sciences, Inc., Farmingdale, NY, USA), anti-BDNF antibody (polyclonal antibody, SC-546, Santa Cruz Biotechnology, Dallas, TX, USA), and anti-β-Actin (monoclonal antibody, SC-47778, Santa Cruz Biotechnology, Dallas, TX). The secondary antibodies used were anti-rabbit and anti-mouse, both of which were HRP-labeled (Perkin-Elmer, Waltham, MA, USA). The protein levels were visualized using a GE ImageQuant image analyzer (LAS-4000) and quantified using the Image J program (Version 1.48).

### Data Analyses

The body-weight data from the four treatment groups were compared using a three-way ANOVA with hormone condition and chronic treatment considered to be between-group factors and the phase of the experiment as a repeated measure (SigmaStat Statistical Software, SYSTAT Software, Inc., Point Richmond, CA, USA). If significant interactions occurred, two-way and one-way ANOVA tests were conducted where appropriate, and these tests were followed by Holm–Sidak *post hoc* tests to determine differences between the treated groups and the untreated control group. Significance was accepted at an α level ≤0.05 for all statistical tests.

Responding in the acquisition and performance components of the multiple-schedule procedure was analyzed in terms of: (1) the overall response rate (total responses/min, excluding timeouts), and (2) the overall accuracy, expressed as the percentage of errors [(incorrect responses/total responses) × 100]. However, when a dose of Δ^9^-THC reduced the overall response rate to less than five responses per minute, the percentage of errors produced by that dose was not calculated due to the small number of responses involved. To determine if there were any statistical differences among the groups in terms of their baseline behavior, data obtained after saline administration (i.e., the control data) were analyzed separately from the drug data using a two-way ANOVA with both hormone status and chronic treatment serving as between-subject factors. Differences in adult sensitivity to Δ^9^-THC or rimonabant were determined by statistically comparing the dose-effect data that resulted from the acute administration of either Δ^9^-THC or rimonabant. For each drug, the dose-effect curves for each treatment group were analyzed using a two-way ANOVA with chronic treatment serving as the between-subjects factor and dose serving as the repeated measure. Significant main effects for dose were analyzed further by Holm–Sidak *post hoc* tests that compared the effects of various doses on response rate and the percentage of errors with the respective control values. Significant interactions were further analyzed by appropriate one-way ANOVA tests for each factor and Holm–Sidak *post hoc* tests.

Western blot quantifications were analyzed by a two-way ANOVA for each protein and each brain region, with hormone status and chronic treatment serving as factors. Significant main effects for each factor were analyzed by Holm–Sidak *post hoc* tests. When a significant interaction occurred, one-way ANOVA tests were conducted on each factor to determine significant differences.

Effects of hormonal status and chronic Δ^9^-THC on behavior were also quantified by comparing the ED50 values and the slopes of the regression lines used to determine the ED50 values. For response rate, the ED50 values represented the estimated dose of Δ^9^-THC that decreased responding from control levels by 50%. For the percentage of errors, the ED50 values represented the estimated dose of Δ^9^-THC that increased the percentage of errors 150%, or 50% above control levels.

## Results

### Effects of Chronic Saline or Δ^9^-THC on Behavioral Training

As shown in **Table 1**, the number of training days did not significantly differ among the treatment groups for each age (adult versus adolescent), as there was no effect of hormone condition [*F*(1,40) = 0.71, *p* > 0.05], chronic treatment [F(1,40) = 0.01, *p* > 0.05], or interactions between any of three experimental factors [age × hormone status: *F*(1,40) = 1.39, *p* > 0.05; age × chronic treatment: *F*(1,40) = 0.5, *p* > 0.05; hormone status × chronic treatment: *F*(1,40) = 2.09, *p* > 0.05; age × hormone status × chronic treatment: *F*(1,40) = 3.68, *p* > 0.05]. However, there was a small, but significant, effect of age on the number of training days [*F*(1,40) = 20.02, *p* < 0.001], with adolescents taking slightly longer to train than adults. This was not surprising in that the chronic Δ^9^-THC injections for more of the adolescents than the adults had to be moved temporarily to the afternoon, as morning injections markedly disrupted training. Nevertheless, the final pattern of responding in the acquisition component had to be characterized by a decrease in the number of errors and an increase in consecutive correct completions of the response sequence each day, as within-session error reduction essentially defined sequence acquisition. There was also a notable difference in the response patterns for the two components, as sequence acquisition was not required in the performance component (i.e., the percentage of errors was typically larger in the acquisition component than in the performance component).

**Table 1:** Mean number of days required to train the subjects under the behavioral procedure during chronic administration.

Treatment group	Adult	Adolescent
Intact/saline	37 ± 2.2^a^	42.5 ± 1.3^b^
Intact/THC	37.2 ± 1.0^a^	45.7 ± 1.9^b^
OVX/saline	36.8 ± 1.5^a^	44.2 ± 1.5^b^
OVX/THC	38.2 ± 1.5^a^	39 ± 1.6^b^
Mean	37.3 ± 0.84	42.8 ± 0.95

*For each treatment group, superscript letters indicate significant differences between ages, but not between hormone status or chronic treatment (e.g., mean group weights designated with a superscript “a” are similar to each other, but significantly different from “b”)*.

### Effects of Chronic Saline or Δ^9^-THC on the Baseline of Behavior

**FIGURE 2:**
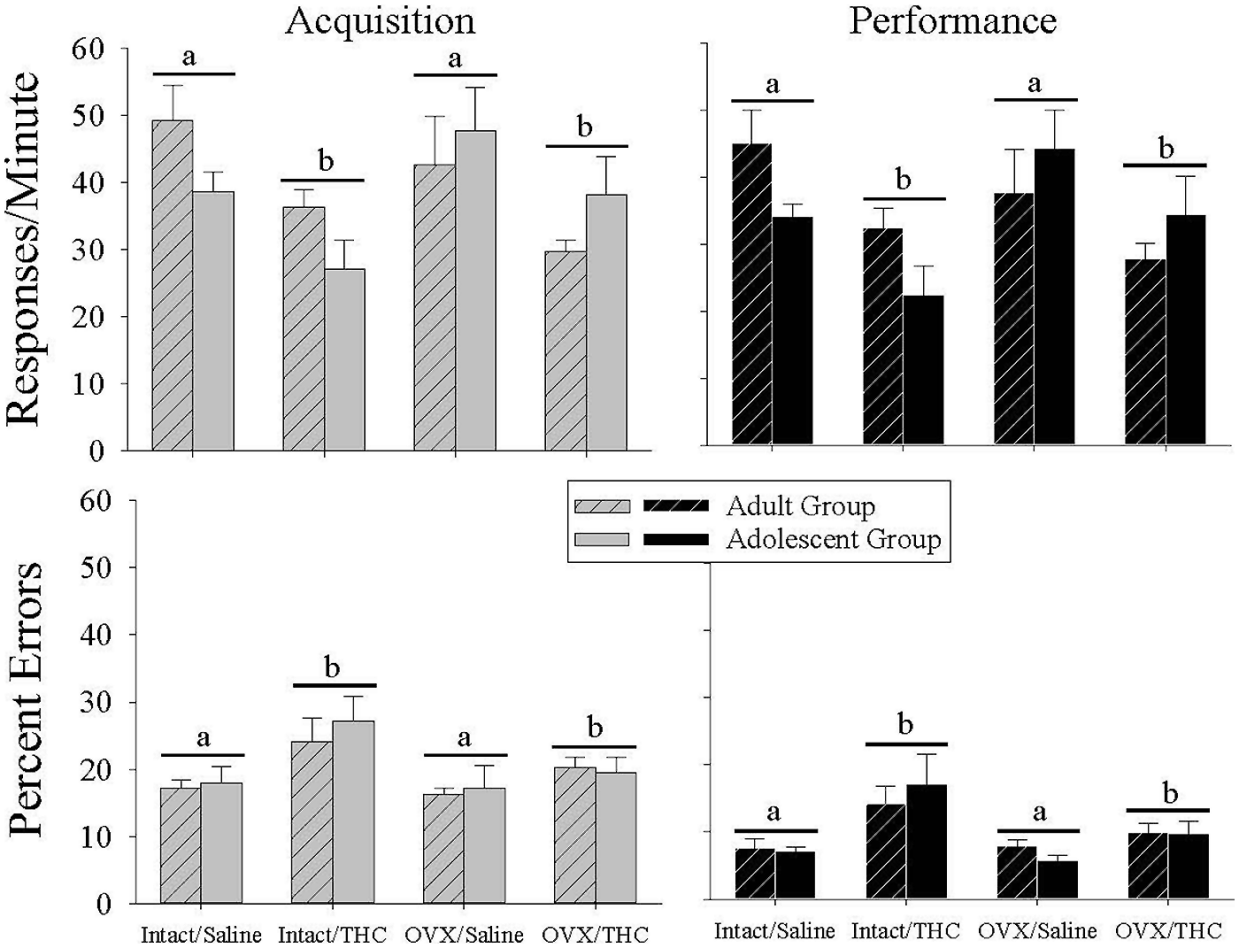
**Bar graphs showing the effects of hormonal status and chronic Δ^9^-THC administration from either early adulthood (*n* = 6/group) or adolescence (*n* = 6/group) on baseline response rates and percent errors under the multiple schedule of repeated acquisition and performance of response sequences**. These data represent the grand mean for 6–20 occasions in each subject when an injection of saline was substituted for the daily chronic dose of Δ^9^-THC. The first of these occasions occurred after a minimum of 65 days after chronic Δ^9^-THC in each age group (early adult or adolescent). As shown by the horizontal bars and letters, there was a significant main effect of chronic Δ^9^-THC on both response rate and percent errors in each component, but no effect of hormone status and no interaction between chronic Δ^9^-THC and hormone status.

The top panels of **Figure 2** show that chronic Δ^9^-THC decreased the response rate irrespective of age in both components of the procedure compared with chronic saline. This was verified statistically by main effects for chronic treatment in the acquisition [*F*(1,40) = 9.48, *p* = 0.004] and performance [*F*(1,40) = 9.65, *p* = 0.003] components. Interestingly, these rate-decreasing effects occurred even though the majority of the chronic injections of saline or Δ^9^-THC were administered during the morning hours and behavioral sessions occurred serially after that time. There were no significant main effects for age [acquisition: *F*(1,40) = 0.16, *p* > 0.05, performance: *F*(1,40) = 0.29, *p* > 0.05] or hormone condition [acquisition: *F*(1,40) = 0.21, *p* > 0.05, performance: *F*(1,40) = 0.52, *p* > 0.05], and no significant interaction for age × chronic treatment [acquisition: *F*(1,40) = 0.11, *p* > 0.05, performance: *F*(1,40) = 0.004, *p* > 0.05] or hormone status × chronic treatment [acquisition: *F*(1,40) = 0.02, *p* > 0.05, performance: *F*(1,40) = 0.11, *p* > 0.05] or age × hormone status × chronic treatment [acquisition: *F*(1,40) = 0.02, *p* > 0.05, performance: *F*(1,40) = 0.007, *p* > 0.05]. There was, however, a significant interaction between age × hormone status in both components [acquisition: *F*(1,40) = 4.85, *p* = 0.03, performance: *F*(1,40) = 5.80, *p* = 0.021], which reflected the general finding that the intact adult groups had higher response rates than the intact adolescent groups, whereas the OVX adult groups had lower response rates than the OVX adolescent groups irrespective of chronic treatment.

With regard to the percentage of errors emitted (**Figure 2**, bottom panels), chronic Δ^9^-THC produced a higher baseline error rate than chronic saline. This was indicated statistically by significant main effects for chronic treatment in the acquisition [*F*(1,40) = 7.75, *p* = 0.008] and performance [*F*(1,40) = 12.18, *p* = 0.001] components, in the absence of significant main effects for age [acquisition: *F*(1,40) = 0.23, *p* > 0.05, performance: *F*(1,40) = 0.00, *p* > 0.05] or hormone status [acquisition: *F*(1,40) = 2.69, *p* > 0.05, performance: *F*(1,40) = 3.86, *p* = 0.056] or of any interactions between these factors in acquisition [age × hormone status: *F*(1,40) = 0.20, *p* > 0.05, age × chronic treatment: *F*(1,40) = 0.003, *p* > 0.05, hormone status × chronic treatment: *F*(1,40) = 1.47, *p* > 0.05, age × hormone status × chronic treatment: *F*(1,40) = 0.26, *p* > 0.05] or performance [age × hormone status: *F*(1,40) = 0.51, *p* > 0.05, age × chronic treatment: *F*(1,40) = 0.71, *p* > 0.05, hormone status × chronic treatment: *F*(1,40) = 2.68, *p* > 0.05, age × hormone status × chronic treatment: *F*(1,40) = 0.03, *p* > 0.05]. Thus, chronic Δ^9^-THC increased errors in the acquisition and performance components similarly in both age groups and in both hormone groups.

### Effects of Δ^9^-THC in Mature Intact and OVX Females Administered Saline Chronically from Early Adulthood (PD 75)

**FIGURE 3:**
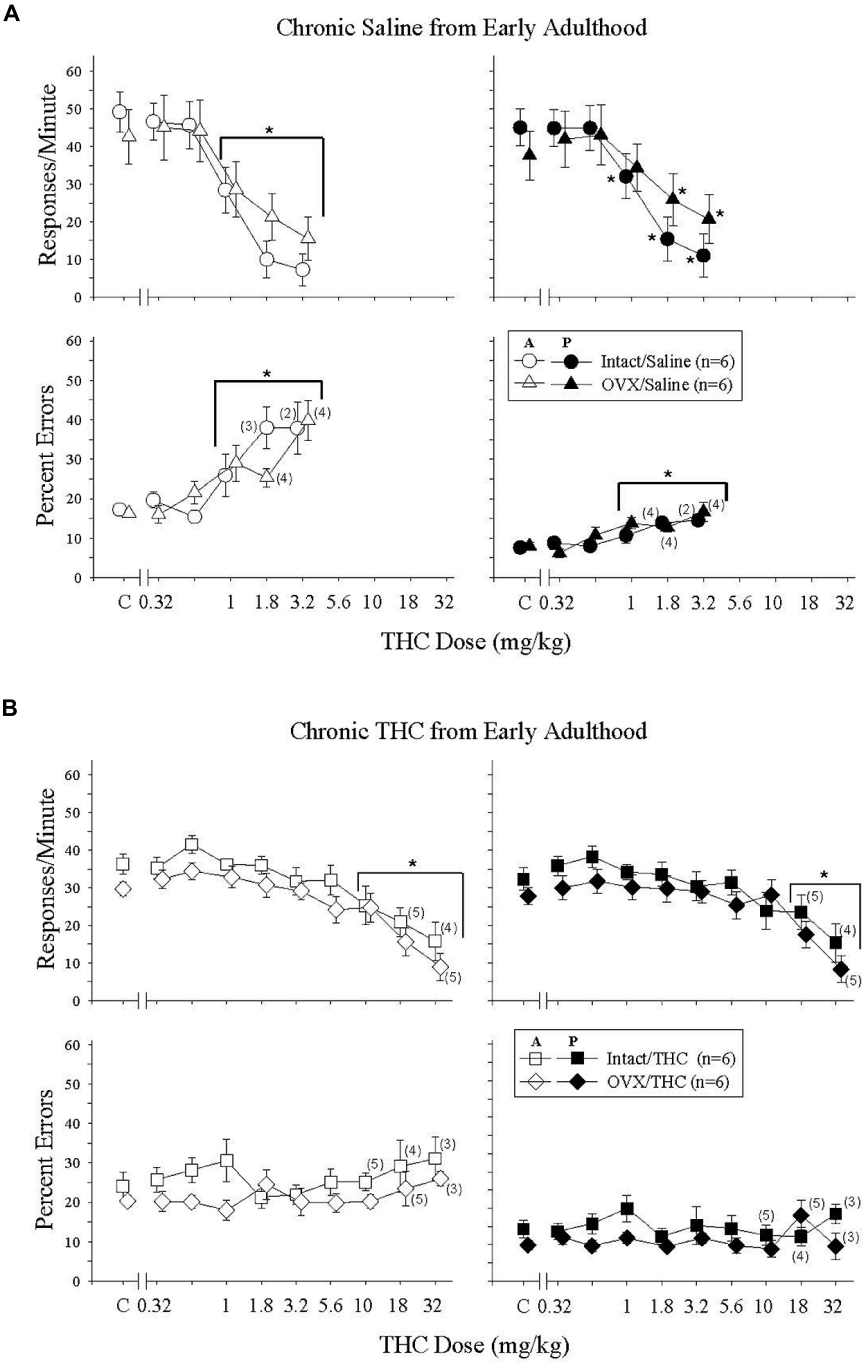
**Acute effects of Δ^9^-THC in intact and OVX females that received either saline (**A**, upper four panels) or 5.6 mg/kg of Δ^9^-THC (**B**, lower four panels) daily from early adulthood to sacrifice and that were responding under an acquisition (A, graph legend) and performance (P, graph legend) procedure.** Data points and vertical lines above C in each panel indicate the grand mean and standard error of the mean (SEM) for 9–19 saline (control) injections. The data points and vertical lines in the dose-effect curves represent a grand mean and SEM for that dose, as each dose was administered on 2–4 occasions in each subject. Asterisks alone or in combination with brackets indicate significant differences between particular doses of Δ^9^-THC and acute saline (control) injections. Numerical values in parentheses and adjacent to a data point indicate the number of subjects represented by that point when it differed from the total number of subjects for that group (e.g., instances in which responding was eliminated entirely and the percentage of errors could not be calculated, or simply differences in the potency of Δ^9^-THC across subjects).

In these Δ^9^-THC-naïve adults, 0.32–3.2 mg/kg of Δ^9^-THC produced dose-dependent rate-decreasing and error-increasing effects in the acquisition and performance components with only modest effects of hormone status evident for response rate in the performance component (**Figure 3A**). These findings were supported by the statistical analyses, which indicated that there was a main effect of Δ^9^-THC dose for both response rate and the percentage of errors in both the acquisition [rate: *F*(5,50) = 52.65, *p* < 0.001; errors: *F*(5,41) = 7.99, *p* < 0.001] and performance [rate: *F*(5,50) = 29.89, *p* < 0.001; errors: *F*(5,42) = 7.38, *p* < 0.001] components, with only a significant interaction between Δ^9^-THC dose and hormone status for response rate in the performance component [*F*(5,50) = 2.60, *p* = 0.036]. Otherwise, there was no significant main effects of hormone status for either dependent measure in the acquisition [rate: *F*(1,50) = 0.03, *p* > 0.05; error: *F*(1,41) = 0.15, *p* > 0.05] and performance [rate: *F*(1,50) = 0.04, *p* > 0.05; error: *F*(1,42) = 0.37, *p* > 0.05] components, and no interaction between hormone status and Δ^9^-THC dose [acquisition: rate, *F*(5,50) = 2.39, *p* = 0.051; error, *F*(5,41) = 1.36, *p* > 0.05, performance: error, *F*(5,42) = 1.16, *p* > 0.05]. In terms of dosages, the 1- to 3.2-mg/kg doses were significantly different from acute saline (control) injections for both response rate and percent errors in the acquisition components and for percent errors in the performance component. Due to the interaction between Δ^9^-THC dose and hormone status for response rate in the performance component, the 1 to 3.2-mg/kg doses were significantly different from acute saline (control) injections for the intact group, but only the 1.8 and 3.2 mg/kg doses were significantly different for the OVX group. Another indication of the effect of hormone status on response rate in the performance component was the greater than twofold shift in the ED50 value for the OVX group compared with the intact group (see **Table 2**).

**Table 2:** Effective Δ^9^-THC dose (i.e., ED50s) for decreasing response rate by 50% and increasing the percentage of errors by 50% in female rats chronically administered saline or Δ^9^-THC from early adulthood or adolescence, as calculated from a linear regression.

ED50 (mg/kg)	Acquisition component				Performance component			
	Resp. Rate	(slope)	Percent error	(slope)	Resp. Rate	(slope)	Percent error	(slope)
**Chronic admin. from adulthood**
Intact/saline	1.24	(-13.38)	0.95	(11.28)	1.57	(-11.84)	1.32	(1.73)
Intact/THC	24.15	(-5.33)	–	(0.29)	>32.0	(-4.84)	–	(-0.06)
OVX/saline	1.97	(-9.31)	0.77	(4.98)	3.89	(-7.55)	1.32	(2.29)
OVX/THC	19.19	(-4.89)	–	(0.55)	22.39	(-9.85)	–	(10.89)
**Chronic admin. from adolescence**
Intact/saline	1.77	(-4.32)	0.77	(5.39)	3.76	(-3.39)	0.60	(4.67)
Intact/THC	29.44	(-2.83)	–	(8.16)	>56.0	(-1.61)	–	(0.16)
OVX/saline	1.62	(-8.19)	0.73	(5.34)	2.83	(-7.44)	0.66	(4.12)
OVX/THC	6.15	(-6.12)	5.12	(10.82)	7.11	(-6.54)	4.40	(9.12)

*A dash (-) indicates values that could not be calculated because a 50% decrease (response rate) or increase (percentage of errors) was not obtained*.

### Effects of Δ^9^-THC in Mature Intact and OVX Females Administered Δ^9^-THC Chronically from Early Adulthood (PD 75)

**Figure 3B** shows the effects of acute Δ^9^-THC in Δ^9^-THC-experienced adults. In these intact and OVX groups, acute Δ^9^-THC produced only dose-dependent rate-decreasing effects in the acquisition and performance components; there were no significant error-increasing effects. Furthermore, these rate-decreasing effects occurred at doses that were 10-folds higher than those that decreased rate in the hormone groups chronically administered saline (compare 3A and 3B, and **Table 2**). All of these observations were supported by the statistical analyses, which indicated that there was a main effect of Δ^9^-THC dose in both behavioral components [acquisition: *F*(9,86) = 16.50, *p* < 0.001, performance: *F*(9,86) = 13.86, *p* < 0.001], but no significant main effect of hormone status [acquisition: *F*(1,86) = 2.04, *p* > 0.05, performance: *F*(1,86) = 0.98, *p* > 0.05] and no significant interaction between these two factors [acquisition: *F*(9,86) = 0.35, *p* > 0.05, performance: *F*(9,86) = 0.74, *p* > 0.05]. The similarity of the effects on response rate across the two hormone groups and across the two behavioral tasks was also evident in the *post hoc* analyses of the grouped data, which found that only the 10–32-mg/kg doses were significantly different from control in the acquisition component, and only the 18–32-mg/kg doses were different from control in the performance component.

The absence of error-increasing effects in the Δ^9^-THC-experienced intact and OVX groups was also verified statistically in that there were no significant main effects for either Δ^9^-THC dose [acquisition: *F*(9,80) = 0.71, *p* > 0.05, performance: *F*(9,80) = 1.24, *p* > 0.05] or hormone status [acquisition: *F*(1,80) = 1.69, *p* > 0.05, performance: *F*(1,80) = 1.67, *p* > 0.05], and no significant interactions between factors [acquisition: *F*(9,80) = 1.22, *p* > 0.05, performance: *F*(9,80) = 1.20, *p* > 0.05] in either component. Based on the near-zero slopes of the dose-effect curves and the fact that none of the doses produced a 50% increase in errors, there are no ED50 values for these groups in **Table 2**.

### Effects of Δ^9^-THC in Mature Intact and OVX Females Administered Saline Chronically from Adolescence (PD 45)

**FIGURE 4:**
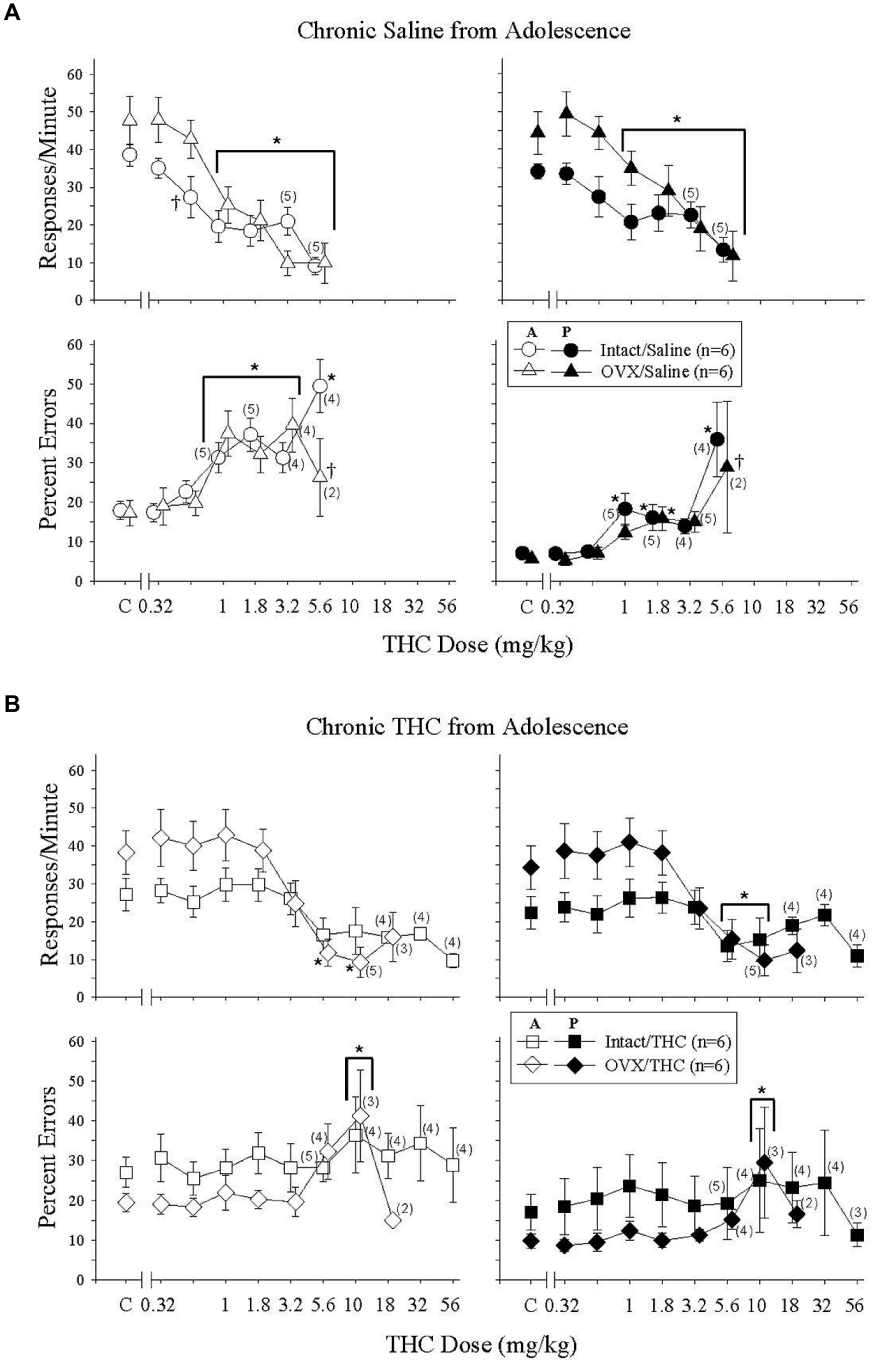
**Acute effects of Δ^9^-THC in intact and OVX females that received either saline **(A)** or 5.6 mg/kg of Δ^9^-THC **(B)** daily from adolescence to sacrifice and that were responding under an acquisition and performance procedure**. Data points and vertical lines above C in each panel indicate the grand mean and SEM for 6–20 saline (control) injections. Asterisks alone or in combination with brackets indicate significant differences between particular doses of Δ^9^-THC and acute saline (control) injections, whereas a cross indicates a significant difference between hormone groups at a particular dose. For additional details, see legend for **Figure 3**.

Acute Δ^9^-THC in these Δ^9^-THC-naïve intact and OVX groups produced similar, but not identical, effects to those in the Δ^9^-THC-naïve hormone groups chronically administered saline from early adulthood (cf. **Figures 3A and 4A**); the difference across these groups experimentally was the age at which chronic saline administration began (PD 75 versus PD 45) and age at which acute Δ^9^-THC was administered to establish these dose-effect curves (PD 141 versus PD 101). The difference in effect was that acute Δ^9^-THC was slightly less potent in these Δ^9^-THC-naïve groups, as almost all of the subjects in each group were able to receive 5.6 mg/kg, and this led to some hormone-dependent variations in the error-increasing effects (i.e., percent errors could only be calculated for 2 of the 6 OVX subjects because their individual response rates were below 5 responses/min).

When the data for response rate were analyzed statistically, there were significant main effects for the dose of Δ^9^-THC in both behavioral components [acquisition: *F*(6,58) = 27.71, *p* < 0.001, performance: *F*(6,58) = 21.25, *p* < 0.001], but no main effects for hormone status [acquisition: *F*(1,58) = 1.15, *p* > 0.05, performance: *F*(1,58) = 2.06, *p* > 0.05]. In the acquisition component, however, there was a significant dose × hormone interaction [*F*(6,58) = 2.26, *p* = 0.05] largely due to a significant difference between hormone groups at the 0.56-mg/kg dose. The interaction was not significant for the responding in the performance component [*F*(6,58) = 2.17, *p* = 0.059]. Regardless of the component, *post hoc* tests indicated that doses of Δ^9^-THC from 1 to 5.6 mg/kg produced decreases in response rate that were significantly different from control injections. Finally, the similarity of the rate-decreasing effects of Δ^9^-THC in these two Δ^9^-THC-naïve hormone groups was reflected in the similarity of the ED50 values for the two groups in each behavioral component (**Table 2**).

The bottom panels of **Figure 4A** show the error-increasing effects produced by Δ^9^-THC in both behavioral components and the significant main effects for dose [acquisition: *F*(6,47) = 12.09, *p* < 0.001, performance, *F*(6,49) = 9.69, *p* < 0.001]. As mentioned above, there were also significant dose × hormone interactions in both components [acquisition: *F*(6,47) = 4.16, *p* = 0.002, performance: *F*(6,49) = 3.48, *p* = 0.006], but no main effects of hormone status [acquisition: *F*(1,47) = 0.43, *p* > 0.05, performance: *F*(1,49) = 2.93, *p* > 0.05]. In the acquisition components, 1–5.6 mg/kg increased errors significantly compared to control in the intact group, whereas 1–3.2 mg/kg increased errors significantly in the OVX group. The 5.6 mg/kg did not increase errors significantly in the OVX group because only one of the two females that were able to respond above 5 responses/min had a marked increase in the percentage of errors. With respect to the percentage errors in the performance component, the 1-, 1.8-, and 5.6-mg/kg doses of Δ^9^-THC increased errors significantly compared to control in the intact group, but only the 1.8-mg/kg dose reliably increased errors significantly in the OVX group. Despite these statistical interactions between Δ^9^-THC dose and hormone status in these Δ^9^-THC-naïve hormone groups, the ED50 values for the percentage of errors in each component were remarkably similar (**Table 2**).

### Effects of Δ^9^-THC for Mature Intact and OVX Females Administered Δ^9^-THC Chronically from Adolescence

These particular Δ^9^-THC-experienced hormone groups were: (1) less sensitive to the rate-decreasing and error-increasing effects of acute Δ^9^-THC than either of the two sets of Δ^9^-THC-naïve hormone groups as expected (**Figures 3A and 4A**), (2) more sensitive than the hormone groups that received Δ^9^-THC chronically from early adulthood (PD 75) onward (**Figure 3B**), and (3) different from each other. Especially evident was the increased sensitivity of the OVX group that initiated chronic Δ^9^-THC during adolescence compared to the sensitivity of the OVX group that initiated chronic Δ^9^-THC during early adulthood (compare **Figures 3B and 4B**). For example, in the OVX group that began Δ^9^-THC during adolescence, 5.6 and 10 mg/kg significantly decreased response rate and 10 mg/kg increased percent errors in both behavioral components. In the OVX group that began Δ^9^-THC during early adulthood, 10 mg/kg produced only a small, but significant, decrease in response rate and there were no error-increasing effects at any dose tested (see **Figure 3B**).

When these intact and OVX groups with Δ^9^-THC experience from adolescence onward were analyzed statistically, there were main effects of dose on both response rate [acquisition: *F*(8,74) = 10.86, *p* < 0.001, performance: *F*(8,74) = 7.73, *p* < 0.001] and percent errors [acquisition: *F*(8,66) = 4.96, *p* < 0.001, performance: *F*(8,66) = 3.87, *p* < 0.001] in both components, and a significant dose × hormone status interaction for response rate in the acquisition component [*F*(8,74) = 2.32, *p* = 0.03]. No other interactions were significant [acquisition/errors: *F*(8,66) = 1.43, *p* > 0.05, performance/rate: *F*(8,74) = 1.92, *p* > 0.05, performance/errors: *F*(8,66) = 1.65, *p* > 0.05], and there were no main effects of hormone status on response rate [acquisition: *F*(1,74) = 1.05, *p* > 0.05, performance: *F*(1,74) = 1.41, *p* > 0.05] or percent errors [acquisition: *F*(1,66) = 1.0, *p* > 0.05, performance: *F*(1,66) = 0.51, *p* > 0.05].

The difference in Δ^9^-THC potency between these Δ^9^-THC-experienced hormone groups was even more evident from the ED50 values than the statistical analyses. For response rate, the respective ED50 values for the intact and OVX groups were 29.44 and 6.15 for the acquisition component and >56 and 7.11 for the performance component. For percent error, the difference in potency was reflected in the fact that Δ^9^-THC did not produce a 50% increase in errors in the intact group, whereas the ED50 values for the OVX groups in the acquisition and performance components were 5.12 and 4.40 mg/kg, respectively (see **Table 2**).

### Effects of Rimonabant in Mature Intact and OVX Females Administered Saline or Δ^9^-THC Chronically from Adolescence or Early Adulthood

**FIGURE 5:**
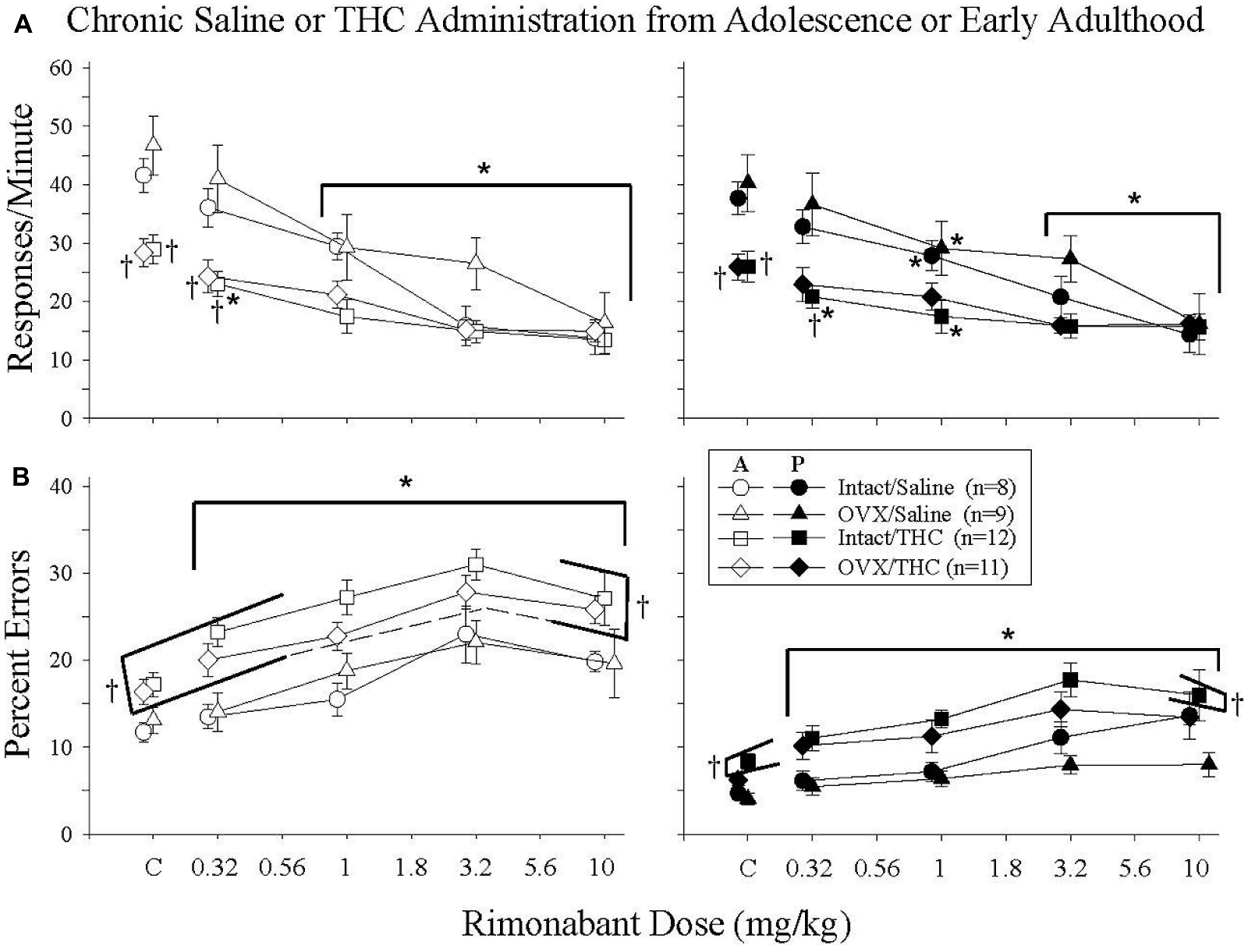
**Acute effects of rimonabant in intact and OVX females that received either saline **(A)** or 5.6 mg/kg of Δ^9^-THC **(B)** daily from adolescence or early adulthood to sacrifice and that were responding under an acquisition and performance procedure**. The data for the two age groups were combined as there was no marked difference between these age groups. Data points and vertical lines above C in each panel indicate the grand mean and SEM for 3–10 vehicle (control) injections administered to each subject in each treatment group. In the upper panels (response rate), asterisks alone or in combination with brackets indicate significant differences between particular doses of Δ^9^-THC and acute saline (control) injections, whereas crosses indicate a significant difference from the intact/saline group under control conditions or after particular doses of rimonabant. In the bottom panels (percent errors), there were no significant interactions, only main effects for dose and treatment group. Therefore, the asterisks with brackets indicate significant differences from control injections for all of the groups at every dose of rimonabant, whereas the crosses with brackets indicate the treatment groups that were significantly different from the intact/saline group irrespective of dose.

Given that separate analyses indicated that there were only minor differences in the effects of rimonabant for the groups that initiated Δ^9^-THC during adolescence and early adulthood, the data for the two ages were combined. As shown in **Figure 5**, rimonabant (0.32–10 mg/kg) produced relatively consistent rate-decreasing and error-increasing effects in all four treatment groups. However, rimonabant affected response rate and percent errors differently. For response rate, differences in the potency of rimonabant’s effects were reflected by significant dose × treatment interactions in both the acquisition [*F*(12,144) = 3.58, *p* < 0.001] and performance [*F*(12,144) = 3.52, *p* < 0.001] components, along with significant main effects of treatment [acquisition: *F*(3,144) = 4.04, *p* = 0.014, performance: *F*(3,144) = 3.24, *p* = 0.033] and rimonabant dose [acquisition: *F*(4,144) = 74.93, *p* < 0.001, performance: *F*(4,144) = 54.19, *p* < 0.001]. In contrast, for percent error, there were no differences in potency among the groups, as there were only main effects of treatment [acquisition: *F*(3,135) = 6.08, *p* = 0.002, performance: *F*(3,138) = 6.78, *p* < 0.001] and rimonabant dose [acquisition: *F*(4,135) = 39.49, *p* < 0.001, performance: *F*(4,138) = 18.82, *p* < 0.001]; the dose × treatment interactions were not significant for responding in either component [acquisition: *F*(12,135) = 0.75, *p* > 0.05, performance: *F*(12,138) = 0.65, *p* > 0.05].

Characterizing the disruptive effects of rimonabant was also complicated by the significant differences in baseline response rates and percent errors for the groups chronically administered saline versus Δ^9^-THC, and by an overall difference in the magnitude of the effects for these groups. For example, the intact/THC group was the only group for which 0.32 mg/kg of rimonabant significantly decreased response rate in both components, yet the dose-effect curves for this group had shallower slopes and larger ED50 values than those for the groups that received saline chronically. In fact, an ED50 value could not be calculated for responding in the performance component because 10 mg/kg of rimonabant did not decrease rate by 50%.

With respect to percent errors, the potency of rimonabant varied little from group to group. This was indicated by the fact that 0.32–10 mg/kg produced significant increases in all of the treatment groups compared to control, and by the similarity of the slopes of the dose-effect curves for each group (**Table 3**). Moreover, due to the similarity of the dose-effect curves for percent errors, there was less of a disparity between the ED50 values and overall magnitude of the effects for each group. As shown in **Table 3**, the rank order of potency with which rimonabant disrupted accuracy in the acquisition components was: intact/THC > OVX/THC > intact/saline > OVX/saline; whereas the rank order of potency with which rimonabant disrupted accuracy in the performance components was: OVX/THC > intact/saline > OVX/saline > intact/THC.

**Table 3:** Effective rimonabant dose (i.e., ED50s) for decreasing response rate by 50% and increasing the percentage of errors by 50% in female rats chronically administered saline or Δ^9^-THC from early adulthood or adolescence, as calculated from a linear regression.

ED50 (mg/kg)	Acquisition component				Performance component			
Treatment group	Resp. Rate	(slope)	Percent error	(slope)	Resp. Rate	(slope)	Percent error	(slope)
Intact/saline	2.48	(-4.03)	0.98	(2.38)	4.20	(-3.12)	0.56	(1.32)
OVX/saline	3.73	(-3.84)	1.46	(2.00)	6.58	(-3.16)	0.59	(0.46)
Intact/THC	4.83	(-1.57)	0.67	(1.95)	–	(-0.85)	0.63	(1.67)
OVX/THC	8.02	(-1.70)	0.76	(1.96)	–	(-1.26)	<0.32	(1.05)

*A dash (-) indicates values that could not be calculated because a 50% decrease (response rate) was not obtained*.

### Effects of Chronically Administered Saline or Δ^9^-THC from Adolescence or Early Adulthood on Hippocampal CB1R, AHA1, and HSP90β in Intact and OVX Females

**FIGURE 6:**
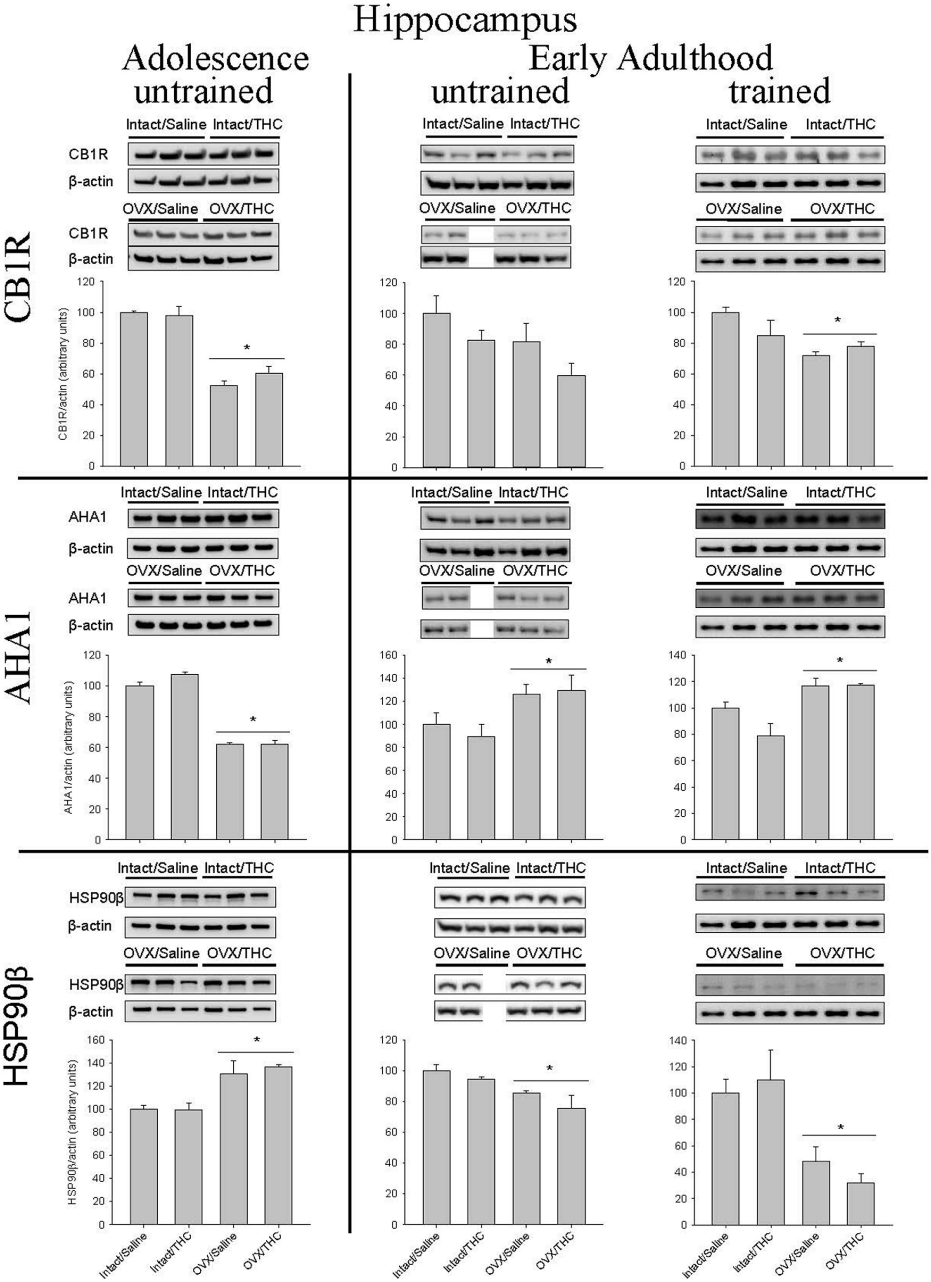
**Effects of chronic Δ^9^-THC and hormone status on the endogenous levels of CB1R, AHA1, and HSP90β in the hippocampus**. Above each bar graph are western blots of protein levels in hippocampal extracts from the female rats in each of the four chronically treated groups. The extracts were separated by 10% SDS-PAGE, transferred onto PDVF membranes, and subjected to western blotting with specific antibodies for each protein. The data were quantified using a GE ImageQuant (LAS-4000 Plus) and the Image J program (Version 1.54). Levels of each protein were corrected for the β-actin levels in the same samples and presented as a ratio of protein to actin in relative units. Asterisks associated with a bar indicate significant main effects of hormone status, as there were no significant main effects of chronic treatment and no significant interaction between chronic Δ^9^-THC and hormone status in hippocampus.

**Figure 6** shows that OVX had a larger effect on hippocampal CB1R levels than chronic Δ^9^-THC administration, regardless of the age at which chronic administration was initiated or behavioral training occurred. For CB1R, this was indicated by significant main effects for hormone status in the groups that were behaviorally naïve (i.e., untrained) from adolescence or early adulthood, and the group that was behaviorally trained during early adulthood [adolescence/untrained: *F*(1,8) = 119.62, *p* < 0.001; adult/untrained: *F*(1,7) = 4.42, *p* > 0.05; adult/trained: *F*(1,11) = 8.91, *p* = 0.012]. There were no significant main effects for chronic treatment [adolescence/untrained: *F*(1,8) = 0.58, *p* > 0.05; adult/untrained: *F*(1,7) = 4.17, *p* > 0.05; adult/trained: *F*(1,11) = 0.62, *p* > 0.05] or chronic treatment × hormone status interactions [adolescence/untrained: *F*(1,8) = 1.76, *p* > 0.05; adult/untrained: *F*(1,7) = 0.06, *p* > 0.05; adult/trained: *F*(1,11) = 3.14, *p* > 0.05].

Similar to CB1R, hippocampal levels of AHA1 were significantly affected by hormone status, but not chronic Δ^9^-THC, and this was again indicated by significant main effects of hormone status in all three groups [adolescence/untrained: *F*(1,8) = 400.72, *p* < 0.001; adult/untrained: *F*(1,7) = 8.67, *p* = 0.022; adult/trained: *F*(1,11) = 18.82, *p* = 0.001]. However, the age at which chronic treatment was initiated produced opposite effects. Compared to the respective intact groups, OVX decreased AHA1 levels in the groups chronically treated from adolescence onward and increased AHA1 levels in both cohorts chronically administered Δ^9^-THC from early adulthood. For all three experimental cohorts, there were no significant main effects of chronic treatment [adolescence/untrained: *F*(1,8) = 3.12, *p* > 0.05; adult/untrained: *F*(1,7) = 0.21, *p* > 0.05; adult/trained: *F*(1,11) = 2.65, *p* > 0.05] and no significant chronic treatment × hormone status interactions [adolescence/untrained: *F*(1,8) = 3.39, *p* > 0.05; adult/untrained: *F*(1,7) = 0.17, *p* > 0.05; adult/trained: *F*(1,11) = 2.86, *p* > 0.05].

The pattern of significant effects for hippocampal HSP90β levels was comparable to that for CB1R and AHA1 levels in that they were all based on hormone status [adolescence/untrained: *F*(1,8) = 24.35, *p* = 0.001; adult/untrained: *F*(1,7) = 9.05, *p* = 0.02; adult/trained: *F*(1,11) = 18.38, *p* = 0.001], but they were inversely related to those for AHA1; that is, compared to the intact groups, HSP90β was increased in the OVX groups chronically treated from adolescence and decreased in both cohorts chronically administered Δ^9^-THC from early adulthood. As was true for all of the hippocampal AHA1 data, there were no significant main effects of chronic treatment [adolescence/untrained: *F*(1,8) = 0.16, *p* > 0.05; adult/untrained: *F*(1,7) = 2.57, *p* > 0.05; adult/trained: *F*(1,11) = 0.05, *p* > 0.05] and no significant chronic treatment × hormone status interactions [adolescence/untrained: *F*(1,8) = 0.24, *p* > 0.05; adult/untrained: *F*(1,7) = 0.34, *p* > 0.05; adult/trained: *F*(1,11) = 0.73, *p* > 0.05].

### Effects of Chronically Administered Saline or Δ^9^-THC from Adolescence or Early Adulthood on Striatal CB1R, AHA1, and HSP90β in Intact and OVX Females

**FIGURE 7:**
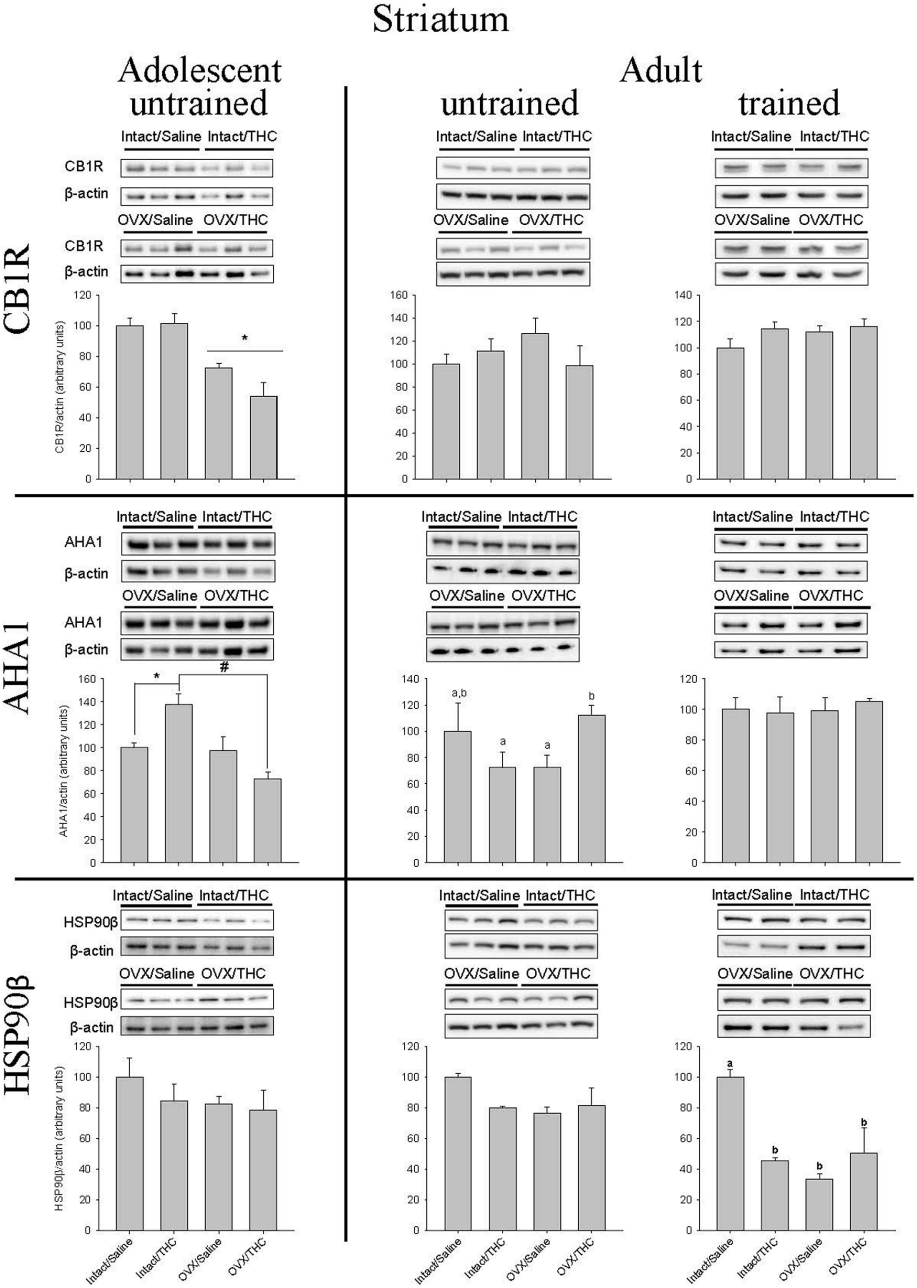
**Effects of chronic Δ^9^-THC and hormone status on the endogenous levels of CB1R, AHA1, and HSP90β in the striatum.** Above each bar graph are western blots of protein levels in striatal extracts from the female rats in each of the four chronically treated groups. Levels of each protein were corrected for the β-actin levels in the same samples and presented as a ratio of protein to actin in relative units. Asterisks associated with a bar indicate significant main effects of hormone status, whereas letters indicate significant differences among the four treatment groups when there were significant main effects for chronic Δ^9^-THC and hormone status or a significant interaction between the factors (e.g., “a” is different from “b,” but not different from “a,b,c”).

The quantification of CB1R, AHA1, and HSP90β protein levels in the striatum yielded substantially different results than those obtained in hippocampus (**Figure 7**). In particular, these proteins were not as affected by hormone status, as only CB1R levels in the groups chronically administered Δ^9^-THC from adolescence were significantly affected by hormone status alone [*F*(1,8) = 35.79, *p* < 0.001; chronic treatment: *F*(1,8) = 1.87; *p* > 0.05; chronic treatment × hormone status: *F*(1,8) = 2.54, *p* > 0.05]. Moreover, there were no significant effects on CB1R in either the untrained [hormone status: *F*(1,7) = 0.03, *p* > 0.05; chronic treatment: *F*(1,7) = 0.08, *p* > 0.05; chronic treatment × hormone status: *F*(1,7) = 1.22, *p* > 0.05] or trained [chronic treatment: *F*(1,12) = 1.88, *p* > 0.05; hormone status: *F*(1,12) = 3.34, *p* > 0.05; chronic treatment × hormone status: *F*(1,12) = 1.13, *p* > 0.05] cohorts that received their chronic treatment from early adulthood onward.

With respect to striatal AHA1 levels, there was a significant chronic treatment × hormone status interaction for the untrained groups from adolescence [chronic treatment × hormone status: *F*(1,8) = 13.44, *p* = 0.006; chronic treatment: *F*(1,8) = 0.55, *p* > 0.05; hormone status: *F*(1,8) = 15.56, *p* = 0.004] and early adulthood [chronic treatment × hormone status: *F*(1,7) = 7.02, *p* = 0.033; chronic treatment: *F*(1,7) = 0.56, *p* > 0.05; hormone status: *F*(1,8) = 0.01, *p* > 0.05], but no significant effect on it in the trained group that received chronic treatment from early adulthood [chronic treatment × hormone status: *F*(1,8) = 0.29, *p* > 0.05; chronic treatment: *F*(1,8) = 0.05, *p* > 0.05; hormone status: *F*(1,8) = 0.18, *p* > 0.05]. However, the pattern of effects for the interactions was different in the untrained groups. In the groups that received chronic treatment from adolescence onward, chronic Δ^9^-THC increased AHA1 levels in the intact group [*F*(1,4) = 12.50, *p* = 0.024], but decreased it in the OVX group [*F*(1,4) = 13.85, *p* = 0.02]. Conversely, in the groups that received chronic Δ^9^-THC from early adulthood onward, there was no effect in the intact females [*F*(1,4) = 1.27, *p* > 0.05] and a relative increase in the OVX females [*F*(1,3) = 26.49, *p* = 0.014].

The effects of chronic treatment and hormone status on HSP90β in striatum did not systematically vary with either AHA1 or CB1R in that there were no significant effects for HSP90β in the untrained groups from adolescence [hormone status: *F*(1,8) = 1.18, *p* > 0.05; chronic treatment: *F*(1,8) = 0.82, *p* > 0.05; chronic treatment × hormone status: *F*(1,8) = 0.28, *p* > 0.05] or early adulthood [hormone status: *F*(1,7) = 2.41, *p* > 0.05; chronic treatment: *F*(1,7) = 1.01, *p* > 0.05; chronic treatment × hormone status: *F*(1,7) = 2.92, *p* > 0.05], but there were significant main effects and a significant chronic treatment × hormone status interaction in the trained adults [hormone status: *F*(1,8) = 12.55, *p* = 0.004; chronic treatment: *F*(1,8) = 4.81, *p* = 0.049; chronic treatment × hormone status: *F*(1,8) = 17.21, *p* = 0.001]. Furthermore, in the groups that were trained behaviorally during early adulthood, the rank order for decreasing HSP90β compared to control was OVX alone [*F*(1,6) = 133.51, *p* < 0.001] ≥ OVX and Δ^9^-THC [*F*(1,6) = 8.64, *p* = 0.026] ≥ chronic Δ^9^-THC alone [*F*(1,6) = 103.87, *p* < 0.001].

### Effects of Chronically Administered Saline or Δ^9^-THC from Adolescence or Early Adulthood on Hippocampal BDNF in Intact and OVX Females

**FIGURE 8:**
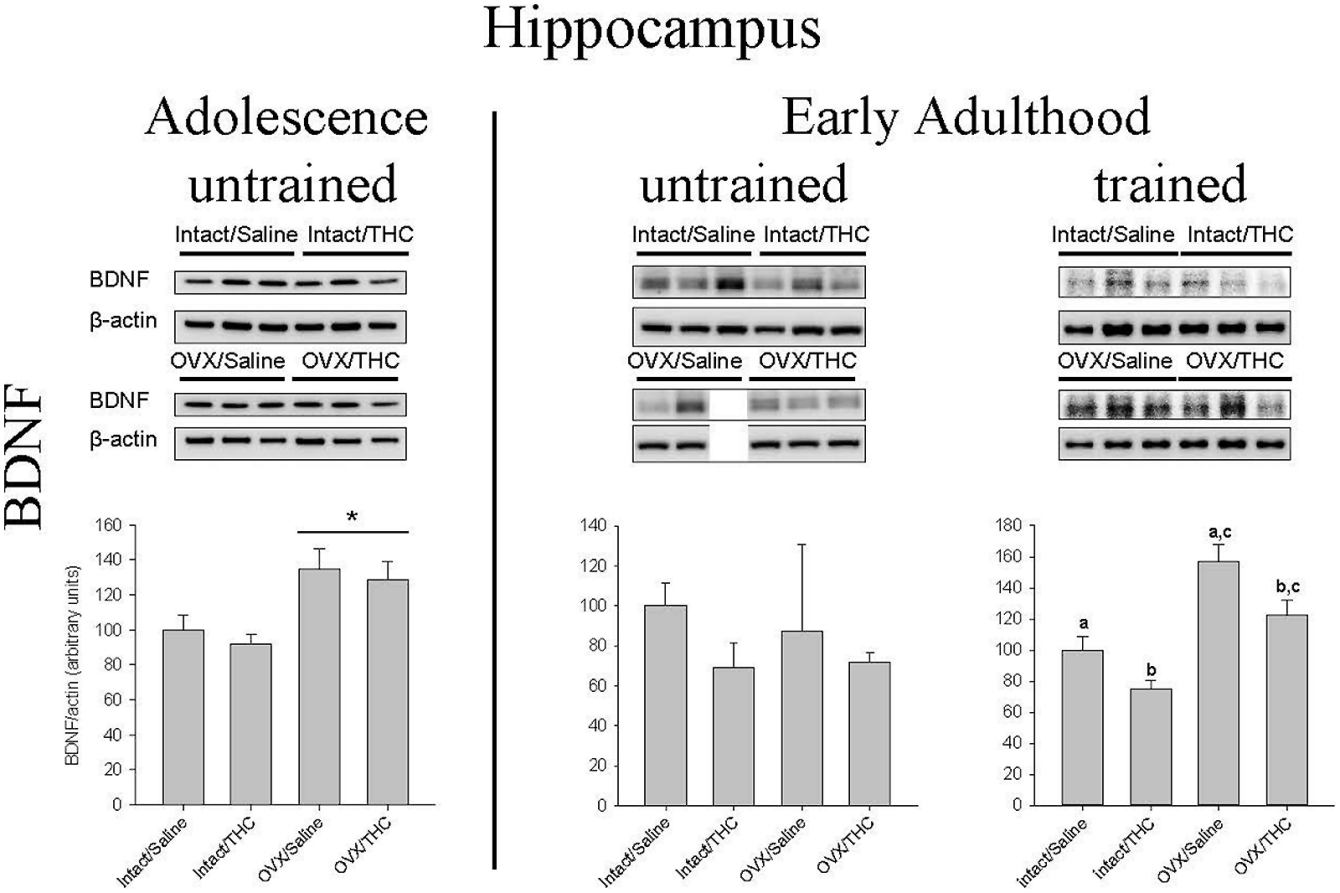
**Effects of chronic Δ^9^-THC and hormone status on the endogenous levels of BDNF in the hippocampus.** Above each bar graph are western blots of protein levels in hippocampal extracts from the female rats in each of the four chronically treated groups. Levels of each protein were corrected for the β-actin levels in the same samples and presented as a ratio of protein to actin in relative units. Asterisks associated with a bar indicate significant main effects of hormone status, whereas letters indicate significant differences among the four treatment groups when there were significant main effects for both chronic Δ^9^-THC and hormone status (e.g., “a” is different from “b,” but not different from “a,c”).

**Figure 8** shows the manner in which hippocampal BDNF was affected by hormonal status, chronic Δ^9^-THC, and age of initiation. In the groups that received chronic Δ^9^-THC from adolescence onward, there was a significant main effect of hormone status [*F*(1,8) = 15.31, *p* = 0.004], but no significant effect of chronic treatment [*F*(1,8) = 0.62, *p* > 0.05] and no hormone status × chronic treatment interaction [*F*(1,8) = 0.01, *p* > 0.05]. In the groups that received their chronic treatments from early adulthood onward, there were no significant effects in the untrained groups [hormone status: *F*(1,7) = 0.08, *p* > 0.05; chronic treatment: *F*(1,7) = 1.80, *p* > 0.05; chronic treatment × hormone status: *F*(1,7) = 0.20, *p* > 0.05], but there were significant main effects of hormone status [*F*(1,11) = 32.57, *p* < 0.001] and chronic treatment [*F*(1,11) = 10.82, *p* = 0.007] in the trained groups; the chronic treatment × hormone status interaction though was not significant [*F*(1,11) = 0.27, *p* > 0.05]. Of particular note for BDNF was the fact that OVX significantly increased BDNF in the untrained groups that initiated their chronic treatment during adolescence, irrespective of the chronic treatment; however, chronic Δ^9^-THC was able to decrease BDNF in both the intact and OVX groups if it was initiated during early adulthood.

### Uterine Weights

Uterine weight was analyzed using a three-way ANOVA, with age (adult versus adolescent), hormone status (intact versus OVX), and chronic treatment (saline versus Δ^9^-THC) serving as factors. This analysis indicated that there was a significant main effect of hormone condition on uterine weight at sacrifice [*F*(1,62) = 126.21, *p* < 0.001], but no main effects for age [*F*(1,62) = 0.002, *p* > 0.05] or chronic treatment [*F*(1,62) = 0.05, *p* > 0.05], and no significant interactions among the three factors [age × hormone status: *F*(1,62) = 0.01, *p* > 0.05, age × chronic treatment: *F*(1,62) = 0.002, *p* > 0.05, hormone status × chronic treatment: *F*(1,62) = 0.5, *p* > 0.05, or age × hormone status × chronic treatment: *F*(1,62) = 0.57, *p* > 0.05]. The mean weight and SEM for the uteri of all of the gonadally intact females (*n* = 23) was 48.2 ± 2.8 mg, whereas the mean weight and SEM for the uteri of the OVX females (*n* = 24) was 10.6 ± 1.5 mg, confirming the effectiveness of the hormone manipulation (i.e., OVX).

## Discussion

Long-term chronic Δ^9^-THC administration initiated during either adolescence or early adulthood consistently disrupted the rate and accuracy with which subjects both acquired new information (sequence acquisition) and emitted well-rehearsed information (sequence performance). This was indicated by main effects of chronic Δ^9^-THC administration on both dependent measures of responding irrespective of hormone status (intact versus OVX) and the age of initiation. However, the age of initiation substantially affected the development of tolerance to its disruptive effects. In subjects that were administered chronic Δ^9^-THC from early adulthood onward, substantial tolerance developed irrespective of hormone status as demonstrated by an approximately 10-fold shift to the right in the Δ^9^-THC dose-effect curves for response rate in both hormone groups, and possibly larger shifts for percent errors. In the subjects that were administered chronic Δ^9^-THC from adolescence onward, the development of tolerance was not as complete, or as extensive, and the OVX group was more sensitive to the rate-decreasing and error-increasing effects of Δ^9^-THC than the intact group. All of these effects also occurred in the absence of dependence, which was assessed by the administration of the CB1R antagonist rimonabant. Furthermore, CB1R protein levels in the hippocampus and striatum were not uniformly affected by chronic Δ^9^-THC. In fact, hormone status produced more significant effects on CB1R, AHA1, and HSP90β levels in hippocampus than chronic Δ^9^-THC treatment; in striatum, the effects varied substantially. Hippocampal BDNF was affected by hormone status more when chronic Δ^9^-THC was initiated during adolescence than when it was initiated during early adulthood.

These types of longitudinal studies are rare and extraordinarily few animals studies have examined the effects of almost life-long Δ^9^-THC, as was the case for the groups that initiated Δ^9^-THC during adolescence. In previous studies from this laboratory, the long-term interactive effects of chronic Δ^9^-THC and ovarian hormones were examined by administering chronic Δ^9^-THC to intact and OVX female rats for some period of time (e.g., 40 days) and then assessing adult sensitivity (Winsauer et al., 2011a). This assessment generally occurred after drug-free training and sensitivity was quantified by comparing the dose-effect curves for the experimental groups (intact/THC, OVX/saline, and OVX/THC) with those of the control group (intact/saline). The age-dependence of these effects was also demonstrated by moving the period of chronic Δ^9^-THC administration from adolescence to early adulthood (Winsauer and Sutton, 2014). In contrast, the aim of the present study was to use the same complex operant procedure as a means of determining whether the age of initiation and hormonal status in females affected the development of tolerance to Δ^9^-THC. Thus, chronic Δ^9^-THC was initiated during either adolescence or early adulthood and continued through behavioral training and the establishment of the dose-effect curves for Δ^9^-THC and the CB1R antagonist rimonabant. In a very pragmatic way, this protocol alone provided direct evidence of the disruptive effects of chronic Δ^9^-THC administration on adolescent development, as comparability between the adolescent groups that received chronic Δ^9^-THC and saline could only be maintained by moving the chronic Δ^9^-THC injections to the afternoons temporarily; and therefore, the training time only differed from the early adult groups by an average of less than 10 days (see **Table 1**). Interestingly, these observations contrast somewhat with data reported in the literature suggesting that adolescent rats may be less sensitive to the behaviorally disruptive effects of Δ^9^-THC than adults (Schramm-Sapyta et al., 2007) and that adolescent females can show greater desensitization of CB1R than adult females and males at both ages (Burston et al., 2010). At least in males, there are also reports that CB1R may be down regulated more readily in adolescents than adult rats (Dalton and Zavitsanou, 2010). Also, Schneider et al. (2008) found that incrementing doses of WIN 55,212-2 over 25 days produced greater effects on object/social recognition, social behavior, play, and self-grooming in pubertal male rats than adult male rats. With respect to the present study, the adolescent groups that were Δ^9^-THC naïve (i.e., chronically administered saline) were less potently affected by acute Δ^9^-THC than the Δ^9^-THC naïve adults.

In many ways, the present results may be summarized best by Barnes and Fried (1974), who stated that ‘degrees of tolerance exist and that the age of the subject at the time of the initial chronic Δ^9^-THC experience interacts with the later development of tolerance to the drug’ (p. 181). Unfortunately, in contrast to the present results, they found that rats that received Δ^9^-THC as adolescents developed tolerance more quickly than their adult counterparts. This was determined in their study by rotorod and balance-beam behavior after 41 days of chronic Δ^9^-THC (4 mg/kg every other day) and a 21-day drug-free period in male rats that were either 30 or 90 days of ages at the start of the chronic regimen. Obviously, there are numerous differences between the two studies, including the type of behavior, the duration of chronic Δ^9^-THC administration, and the insertion of a drug-free period, but the main difference may be the use of male versus female rats. This is not to say that these other variables are not important, but that the long-term behavioral effects resulting from chronic cannabinoids during adolescence are frequently different in males and females (Biscaia et al., 2003; Mateos et al., 2011; Realini et al., 2011; Lee et al., 2014), and that adolescence, as opposed to adulthood, is a particularly vulnerable time for females (O’Shea et al., 2006; Cha et al., 2007; Winsauer et al., 2011a) and males (Stiglick and Kalant, 1983, 1985; Schneider and Koch, 2003; Cha et al., 2006).

Along with the unexpected sensitivity of the adolescent groups, the present data indicated that: (1) the initiation of chronic Δ^9^-THC during early adulthood eliminated differences in the development of tolerance between intact and OVX females, and (2) the sensitivity of the OVX groups was increased relative to the intact groups if chronic Δ^9^-THC administration was initiated during adolescence. The first result was unexpected because recent data from our laboratory indicated that a period of chronic administration during early adulthood (PND 75–115) did not alter the hormonally dependent effects of Δ^9^-THC. In other words, intact female rats were more sensitive than OVX females to the rate-decreasing and error-increasing effects of Δ^9^-THC regardless of when a 40-days period of chronic Δ^9^-THC was initiated (adolescence or early adulthood). However, in the present study, there was no main effect of hormone status or a significant interaction between hormone status and chronic Δ^9^-THC administration for either dependent measure if it was initiated during early adulthood. In fact, the dose-effect curves for both the intact and OVX groups were remarkably similar, as was the magnitude of the level of tolerance. These data would suggest that long-term chronic Δ^9^-THC can antagonize the effects of ovarian hormones on acquisition and performance behavior, and support existing data indicating that the interaction between the sex hormones and cannabinoids or endocannabinoids is bidirectional (for reviews, see Lopez, 2010; Viveros et al., 2011; Gorzalka and Dang, 2012; Craft et al., 2013).

The second long-term effect of uninterrupted chronic Δ^9^-THC from adolescence onward was the relative increase in the sensitivity of the OVX females compared to intact females. As mentioned above, we found OVX females to be less sensitive to Δ^9^-THC than intact females in two studies (Winsauer et al., 2011a; Winsauer and Sutton, 2014), irrespective of the age at which the 40 days of chronic Δ^9^-THC administration was initiated (i.e., adolescence or early adulthood). The only difference between these two studies and the present study was that both of the prior studies had a substantial drug-free period after the 40 days of Δ^9^-THC, but prior to the acute dose-effect determinations, for behavioral training. This would suggest that some of the organizational and activational effects of the ovarian hormones likely occur during this drug-free period, and as a result these intact females have an increased sensitivity to subsequent cannabinoid administration compared with an OVX female. Conversely, uninterrupted chronic Δ^9^-THC from adolescence onward must prevent some of the organizational effects of the ovarian hormones. If this is the case, this may be some of the first evidence (to our knowledge) that suggests that chronic Δ^9^-THC may oppose the organizational effects of the ovarian hormones, as well as their activational effects (Craft et al., 2013). Exactly which organizational effects is difficult to say, but clearly the reduced sensitivity of the intact group compared to the OVX group after chronic administration in adolescents (particularly at higher doses) is something that could have long-term behavioral ramifications.

Although the effects of OVX on the pharmacokinetic (metabolic) disposition of Δ^9^-THC in females was not examined in this study, there is evidence that male and female rats metabolize Δ^9^-THC differently via the P450 system. Females produce more of the active metabolite 11-hydroxy-Δ^9^-tetrahydrocannabinol (11-OH-Δ^9^-THC), whereas males produce more inactive metabolites (Narimatsu et al., 1991). Thus, female rats likely have more active metabolites in the brain after both acute and chronic Δ^9^-THC administration (Tseng et al., 2004; Wiley and Burston, 2014). In one study, for example, concentrations of 11-OH-Δ^9^-THC in the brain tissue of females peaked at 30 and 120 min and then declined to near zero levels by 4 h, as compared with brain tissue concentrations in males that reached a lower maximal peak by 30 min and then declined to near zero levels by 2 h (Tseng et al., 2004). Whether the metabolic differences between females and males has some similarity to differences between intact and OVX females, due to the loss of hormone in the OVX females, is not known. Nevertheless, such a dispositional difference would not easily explain the increased sensitivity of OVX females chronically treated with Δ^9^-THC during adolescence.

As in previous animal studies where chronic administration of Δ^9^-THC may have led to dependence (Aceto et al., 1996; Cook et al., 1998; Delatte et al., 2002), the level of dependence was assessed by the administration of varying doses of the CB1R antagonist rimonabant (or SR141716A as it was formerly known). The major advantage of administering an antagonist over simply terminating Δ^9^-THC administration for some period of time was that a precipitated withdrawal often elicits a larger number of withdrawal-associated effects, and they generally occur over a more predictable time frame and with greater intensity, which makes quantifying these effects easier. Among some of the many withdrawal-associated effects reported for Δ^9^-THC are: scratching, facial rubs, licking, wet-dog shakes, paw shakes, retropulsion, head shakes, forepaw treading, and ptosis (Aceto et al., 1996). In behavioral studies involving operant procedures, the disruption of food-maintained responding has also served as a means for assessing dependence on Δ^9^-THC. In fact, disruptions of schedule-controlled responding have been posited to be a more sensitive indicator of dependence than many of the aforementioned gross behavioral effects. For example, both Slifer et al. (1984) and Beardsley et al. (1986) found that reductions in rates of operant responding during withdrawal from Δ^9^-THC and phencyclidine, respectively, occurred more reliably than dramatic changes in gross behavior and often took longer to return to control levels after withdrawal of Δ^9^-THC than gross behavior. In the present study, 0.32–10 mg/kg of rimonabant disrupted response rate and accuracy with similar potency in both the saline- and Δ^9^-THC-treated groups, and based on this similarity there was little or no evidence that significant dependence had developed during chronic Δ^9^-THC administration. Both the saline- and Δ^9^-THC-treated females were, however, more sensitive to the effects of rimonabant than the males from a previous study (Delatte et al., 2002). In that study, 1 mg/kg did not disrupt either response rate or percent errors prior to chronic Δ^9^-THC administration. These data support the long-standing supposition that dependence is a dose-dependent phenomenon (Jones et al., 1976), and that it may, or may not, be evident with the development of tolerance.

Because OVX occurred at the same time for both the adolescent and early adult groups, and because there was no significant difference in tolerance between the intact and OVX groups from early adulthood, the reduced magnitude of tolerance in the adolescent groups point to an age-dependent effect. In other words, even OVX females were able to develop substantial tolerance if they were allowed to age for some drug-free period, which suggests the endocannabinoid system (like the endocrine system) and its interaction with the HPG/HPA axes (Lopez, 2010) must also be allowed to mature or function unperturbed to develop properly. Furthermore, at least in hippocampus, the absence of a main effect of chronic Δ^9^-THC on CB1R suggests that it may well be the downstream signaling mechanisms that require time for development (e.g., Ras/ERK signaling Rubino et al., 2005). This latter suggestion is supported by the fact that hormone status had completely opposite effects on the relative expression levels of AHA1 and HSP90β in the hippocampus when the age of chronic Δ^9^-THC administration differed. We previously demonstrated that AHA1, a co-activator of HSP90, specifically upregulates the trafficking and signaling of CB1R (Filipeanu et al., 2011) and the present data suggest that subtle changes in the levels of AHA1 could alter CB1R subcellular localization, and thus, the response to Δ^9^-THC administration. Interestingly, the present hippocampal data also show an inverse relationship between AHA1 and HSP90β expression that was not seen previously with cerebellar tissue from adults that received the drug for 40 days as adolescents (Filipeanu et al., 2011). Whether this relationship is unique to the hippocampus or is the result of the uninterrupted regimen of chronic Δ^9^-THC remains to be demonstrated, but there is little doubt that expression levels may be under the control of multiple factors and that uninterrupted chronic Δ^9^-THC from adolescence onward can produce brain-region-specific effects that are different from those that occur after a drug-free maturation period.

Unlike the effects of chronic Δ^9^-THC and hormone status on CB1R signaling proteins in hippocampus, the effects of these variables in striatum varied across ages and the specific protein. For instance, despite significant tolerance, there was no CB1R downregulation in either hormone group administered chronic Δ^9^-THC from early adulthood onward and there was no readily discernable pattern of effects on AHA1 or HSP90β in the hormone groups from either age of initiation. These data indicate that the expression, subcellular localization and signaling of CB1R and its co-chaperones are regulated in a brain-region specific manner in response to chronic Δ^9^-THC. Classically, CB tolerance has been tied directly to reversible and regionally selective downregulation of CB1R in humans (Villares, 2007; Hirvonen et al., 2012) as well as animals (Oviedo et al., 1993; Fan et al., 1996; Romero et al., 1998). However, more recent studies with various cannabinoid agonists have provided evidence that behavioral tolerance can occur with CB1R desensitization alone (Rubino et al., 2000a; Daigle et al., 2008), or a region-dependent balance of desensitization and downregulation (Breivogel et al., 1999; McKinney et al., 2008). Two data-driven suppositions that have also been advanced are: (1) desensitization and downregulation likely have different agonist-dependent time courses (Wu et al., 2008), and (2) desensitization likely occurs before downregulation (Rubino et al., 2000a). Age-dependent differences in expression levels of CB1 mRNA have even been found within the different regions of striatum (Van Waes et al., 2012), and this region has been shown to adapt more slowly to chronic CB than hippocampus or cerebellum (Romero et al., 1998; Zhuang et al., 1998; Breivogel et al., 1999; McKinney et al., 2008). Finally, in what could explain some of our results from the present study, Zhuang et al. (1998) suggested that CB1R expression (or at least mRNA for CB1R) may be able to return to control levels after a period of time even if increases in the level of receptor agonists are sustained. In the striatum in particular, tolerance to the hypomotility produced by chronic Δ^9^-THC was proposed to depend more on Ras/ERK signaling than either downregulation or desensitization (Rubino et al., 2005).

As was true for AHA1 and HSP90β expression in hippocampus, the effects of chronic Δ^9^-THC administration on hippocampal BDNF differed depending on the age of initiation. BDNF is known as an important signaling molecule that is affected by chronic cannabinoids in a sex- and age-dependent manner (Lopez-Gallardo et al., 2012). In the present study, when chronic Δ^9^-THC was initiated during adolescence, it had little or no effect on adult BDNF expression as it was significantly increased only in the treated and untreated OVX females. This is consistent with findings from Riebe et al. (2010), who also postulated that the upregulation of CB1R densities occurred in the OVX females because estradiol typically inhibits CB1R expression. Interestingly, they also postulated that an upregulation of hippocampal CB1R densities could occur as a compensatory response to Δ^9^-THC’s capacity to produce desensitization both acutely (Mize and Alper, 2000; Wu et al., 2008) and chronically (Rubino et al., 2000c; Daigle et al., 2008). If correct, this mechanism could also help explain why CB1R densities were increased even in the OVX females that were chronically administered Δ^9^-THC from adolescence onward.

When chronic Δ^9^-THC was initiated during early adulthood, BDNF expression was decreased in both intact and OVX females compared to controls (see **Figure 8**), although this was only a trend for the untrained females. Nevertheless, these data are also consistent with the existing literature because they show the capacity of chronic Δ^9^-THC to decrease BDNF in hippocampus (e.g., Derkinderen et al., 2003), an area integrally involved in learning and memory (Lichtman et al., 1995; Wise et al., 2009; Abush and Akirav, 2010). This would also seem to be an effect that is readily opposed by estradiol in the hippocampus (Scharfman and Maclusky, 2005; Wallace et al., 2006). For some time, there was evidence that chronic Δ^9^-THC altered hippocampal morphology in ‘young adult’ male rats (e.g., Landfield et al., 1988; Lawston et al., 2000), but scientists had not worked out all of the biochemical pathways that can lead to neuro- and synaptogenesis, and there was a true lack of data regarding these processes in females. Moreover, researchers are only now beginning to understand all of the complexities involved in neural and synaptic plasticity in the hippocampus, and the profound role estrogens have on the structure and function of the hippocampus during pubertal development (Sisk and Zehr, 2005) and throughout females’ lives (Adams et al., 2001; also see Spencer et al., 2008, for a review). Some of the more recent studies have also established a clear brain-region-dependent interaction between BDNF and CB1R (Butovsky et al., 2005; De Chiara et al., 2010). For example, chronic prenatal administration of WIN 55,212,2 has been shown to produce significant reductions in BDNF protein levels in cytoplasmic fractions from hippocampus and frontal cortex of adult rats (Maj et al., 2007), but chronic Δ^9^-THC for 7 days produced a clear up-regulation of BDNF mRNA and protein in nucleus accumbens, ventral tegmental area, and mPFC (Butovsky et al., 2005). Unfortunately, these studies also show the extent to which chronic Δ^9^-THC, hormone status, brain region, and age can interact to affect neural and synaptic plasticity, and that substantial research will be needed to fully understand their interaction as it relates to acquisition and performance behavior.

In summary, the present data suggest that the capacity of females to become tolerant to the disruptive effects of chronic Δ^9^-THC on acquisition and performance behavior can differ markedly depending on the presence of ovarian hormones and the age of initiation in females. This was evident from the data indicating that: (1) tolerance developed to a much lesser extent when chronic Δ^9^-THC was initiated in adolescence versus early adulthood, and (2) adolescent initiation of chronic Δ^9^-THC markedly reduced the sensitivity of intact females and increased the sensitivity of OVX females – an effect that did not occur when chronic Δ^9^-THC was initiated in early adulthood (e.g., Winsauer and Sutton, 2014) or when a drug-free period followed 40 days of chronic Δ^9^-THC during adolescence (Winsauer et al., 2011a). Together, these data suggest that a drug-free period may be necessary for both the endocrine and endocannabinoid systems to develop properly and exert their usual influence on behavior. The age- and region-dependence of the interaction between hormone status and chronic Δ^9^-THC was also evident from the expression of CB1R, AHA1, and HSP90β. In the hippocampus of females, chronic Δ^9^-THC could not obscure the predominant effect of hormone status on CB1R expression regardless of the age of initiation; however, age of initiation resulted in contrasting levels of adult expression of AHA1 and HSP90β. In striatum, chronic Δ^9^-THC could not obscure the influence of hormone on adult CB1R expression if it was initiated during adolescence, and had no long-term effect on CB1R if it was initiated during early adulthood. The effects on AHA1 and HSP90β in striatum were mixed. Finally, the effects of chronic Δ^9^-THC on adult hippocampal BDNF were obscured by hormone status if administration began during adolescence, but BDNF was decreased by it if administration began in early adulthood. Taken together, these data indicate that chronic Δ^9^-THC from adolescence onward limits the development of behavioral tolerance compared with chronic Δ^9^-THC later in life, and may reduce the long-term responsiveness of the CB system in various brain regions critical for learning and memory.

## Author Contributions

PJW and JS participated in the ovariectomies, collection all of the behavioral data, and brain dissections. CF and PFW were responsible for the Western blot analyses. All of the authors contributed to the writing of the paper by critically reviewing content and approving of the final version for publication.

## Conflict of Interest Statement

The authors declare that the research was conducted in the absence of any commercial or financial relationships that could be construed as a potential conflict of interest.
